# Digital Health Psychosocial Intervention in Adult Patients With Cancer and Their Families: Systematic Review and Meta-Analysis

**DOI:** 10.2196/46116

**Published:** 2024-02-05

**Authors:** Yingzi Zhang, Marie Flannery, Zhihong Zhang, Meghan Underhill-Blazey, Melanie Bobry, Natalie Leblanc, Darcey Rodriguez, Chen Zhang

**Affiliations:** 1 Magnet Program and Nursing Research Department UT Southwestern Medical Center Dallas, TX United States; 2 School of Nursing University of Rochester Medical Center Rochester, NY United States; 3 Edward G Miner Library University of Rochester Medical Center Rochester, NY United States

**Keywords:** cancer, anxiety, decision-making, depression, digital health, distress, family, mental health, mortality, psychosocial intervention, quality of life

## Abstract

**Background:**

Patients with cancer and their families often experience significant distress and deterioration in their quality of life. Psychosocial interventions were used to address patients’ and families’ psychosocial needs. Digital technology is increasingly being used to deliver psychosocial interventions to patients with cancer and their families.

**Objective:**

A systematic review and meta-analysis were conducted to review the characteristics and effectiveness of digital health interventions on psychosocial outcomes in adult patients with cancer and their family members.

**Methods:**

Databases (PubMed, Cochrane Library, Web of Science, Embase, CINAHL, PsycINFO, ProQuest Dissertations and Theses Global, and ClinicalTrials.gov) were searched for randomized controlled trials (RCTs) or quasi-experimental studies that tested the effects of a digital intervention on psychosocial outcomes. The Joanna Briggs Institute’s critical appraisal checklists for RCTs and quasi-experimental studies were used to assess quality. Standardized mean differences (ie, Hedges *g*) were calculated to compare intervention effectiveness. Subgroup analysis was planned to examine the effect of delivery mode, duration of the intervention, type of control, and dosage on outcomes using a random-effects modeling approach.

**Results:**

A total of 65 studies involving 10,361 patients (mean 159, SD 166; range 9-803 patients per study) and 1045 caregivers or partners (mean 16, SD 54; range 9-244 caregivers or partners per study) were included in the systematic review. Of these, 32 studies were included in a meta-analysis of the effects of digital health interventions on quality of life, anxiety, depression, distress, and self-efficacy. Overall, the RCT studies’ general quality was mixed (applicable scores: mean 0.61, SD 0.12; range 0.38-0.91). Quasi-experimental studies were generally of moderate to high quality (applicable scores: mean 0.75, SD 0.08; range 0.63-0.89). Psychoeducation and cognitive-behavioral strategies were commonly used. More than half (n=38, 59%) did not identify a conceptual or theoretical framework. Most interventions were delivered through the internet (n=40, 62%). The median number of intervention sessions was 6 (range 1-56). The frequency of the intervention was highly variable, with self-paced (n=26, 40%) being the most common. The median duration was 8 weeks. The meta-analysis results showed that digital psychosocial interventions were effective in improving patients’ quality of life with a small effect size (Hedges *g*=0.05, 95% CI –0.01 to 0.10; *I*^2^=42.7%; *P*=.01). The interventions effectively reduced anxiety and depression symptoms in patients, as shown by moderate effect sizes on Hospital Anxiety and Depression Scale total scores (Hedges *g*=–0.72, 95% CI –1.89 to 0.46; *I*^2^=97.6%; *P*<.001).

**Conclusions:**

This study demonstrated the effectiveness of digital health interventions on quality of life, anxiety, and depression in patients. Future research with a clear description of the methodology to enhance the ability to perform meta-analysis is needed. Moreover, this study provides preliminary evidence to support the integration of existing digital health psychosocial interventions in clinical practice.

**Trial Registration:**

PROSPERO CRD42020189698; https://www.crd.york.ac.uk/prospero/display_record.php?RecordID=189698

## Introduction

Cancer is often associated with psychological distress in patients and their family members. Emerging evidence shows that psychological distress contributes to cancer mortality [1,2]. Given that over 2 million new cancer cases are expected to be diagnosed in 2024 in the United States, psychosocial distress is a significant public health problem [3]. Psychosocial distress can be triggered by many challenges, such as decision-making regarding treatment, self-care challenges due to side effects from cancer treatment, maintaining work-life balance, and financial burden. A large body of research documents the negative influence of a cancer diagnosis and treatment on a patient’s experience, including depression, anxiety, and decreased quality of life [4,5]. Cancer not only affects the patient but also imposes changes on the family [6]. Family members, who often assume caregiving roles to complement the roles of the health care team, often experience deteriorating quality of life and significant psychological distress [7,8]. For many years, researchers have examined psychosocial interventions addressing patients’ and family members’ needs to help maintain psychosocial well-being and quality of life during the cancer experience [9-12].

Increasingly, studies have used digital technology to deliver psychosocial interventions. In this report, we refer to digital health intervention as the use of digital, mobile, and wireless technologies to deliver an intervention. Digital health interventions have gained popularity due to their geographic accessibility, self-paced nature, user-friendly design, up-to-date information provision, and time-sensitive interaction with health care providers [13,14]. Further, digital interventions have significant potential for reaching people, mainly in rural areas or people with limited mobility [15]. There are various delivery modes for digital interventions, such as smartphone apps, websites, the internet, and virtual reality. There are also drawbacks, including concerns related to security and privacy and inaccessibility for people without smart device ownership. Psychosocial interventions may incorporate various components, such as communication skills training, cognitive behavioral therapy, patient education, peer support, and problem-solving training [16].

Despite the plethora of individual research studies, a synthesis of digital psychosocial interventions for patients with cancer and their families is needed to provide a summary of existing evidence regarding the effects of interventions and provide directions for future research and clinical practice. A range of systematic reviews have examined digital health psychosocial interventions for patients with cancer [17-22] and their family members [23,24]. However, these reviews have limitations. For example, some reviews primarily focused on a specific population, such as individuals with breast [17] or prostate cancer [19,20]; a particular delivery mode, such as internet-based [17,23,24]; or a specific psychosocial outcome, such as quality of life or psychological distress [21,22]. In addition, Slev et al [25] synthesized evidence from systematic reviews of interventions delivered through computers or the internet for patients with cancer and their caregivers; however, the authors failed to quantify the effectiveness of interventions across studies using advanced statistical techniques, such as a meta-analysis. To date, no studies have used meta-analytical strategies to quantify the impact of digital health interventions on psychosocial outcomes in patients with cancer and family members. To fill these gaps, we conducted a systematic review and meta-analysis to comprehensively review the characteristics and effectiveness of digital psychosocial interventions on psychosocial outcomes across different available delivery modes in adult patients with cancer and their family members.

The specific aims were to answer the following questions:

1. What are the characteristics of digital psychosocial interventions for adult patients and families living with cancer? (ie, intervention component, theoretical or conceptual framework, tailored or standardized, mode of delivery, prescribed dosage, duration of the intervention, and actual dosage)?
2. What is the efficacy of interventions on psychosocial outcomes for adult individuals diagnosed with cancer and their family members and associated factors (ie, delivery mode, control condition, and dosage, including the number of sessions, frequency, and duration)?

## Methods

The review followed the PRISMA (Preferred Reporting Items for Systematic Reviews and Meta-Analyses) checklist [26].

### Study Identification

The medical librarian (DR) and first author (YZ) worked together to identify search terms to build a comprehensive search strategy (Multimedia Appendix 1). Using controlled vocabulary and keywords when available, the search strategy was executed in the following databases: PubMed, Cochrane Library, Web of Science, Embase, CINAHL, PsycINFO, ProQuest Dissertations and Theses Global, and ClinicalTrials.gov. The results were limited to the English language and those published from each resource’s inception until March 2019, when the search was completed. An initial limited search of PubMed and CINAHL was undertaken, followed by an analysis of the text words in the abstract and the index terms used to describe the article. Relevancy was determined by the first author (YZ) and medical librarian (DR). A second search was undertaken across all included databases using all identified keywords and index terms.

### Study Selection

The inclusion criteria were studies that (1) included adult patients (≥18 years of age with any cancer diagnosis) or their adult family members (eg, partner, caregiver, adult children, parent, or relative); (2) tested a digital health psychosocial intervention, which was defined as any nonpharmacological therapeutic intervention that addressed the psychological, social, personal, or relational adjustment needs associated with cancer through a digital health mechanism (eg, application and website); (3) measured at least 1 psychosocial outcome; and (4) used an experimental (randomized controlled trial [RCT]) or a quasi-experimental design. Studies were excluded if they enrolled pediatric patients with cancer; were review articles, letters to the editor, editorial reports, case reports, or commentaries; were published as abstracts only; and were not published in English. For meta-analysis, we excluded articles that did not provide data or when only a single study included the outcome measure.

After removing duplicates, the first author (YZ) read all titles and abstracts to identify articles based on inclusion and exclusion criteria. The full texts of all included articles were then screened independently by 2 reviewers (master’s-level or above), and final decisions were made based on consensus. Finally, articles identified in the search were imported to Endnote X8 (Clarivate Analytics).

### Data Extraction and Management

A Microsoft Excel (Microsoft Corporation) spreadsheet was used to record information [27], including the description of the interventions (eg, theory basis, mode of delivery, content, actual dosage, planned dosage, standardized, or tailored), study sample (eg, age, sex, education, race, ethnicity, and cancer diagnosis), study characteristics (eg, design, randomization method, and control condition), intervention outcome variables and measurements, follow-ups, and quantitative data (ie, mean, SD, and sample size). Dosage was described as the number of intervention sessions, frequency, and duration of access to intervention. A standardized intervention was defined as all participants receiving the same intervention, while a tailored intervention involved customization of the intervention based on individual characteristics or needs [28]. We defined the prescribed dosage as the intended treatment dose, including the number of intervention sessions, frequency, and total length according to the study protocol. A codebook was created for data extraction, and the team’s decisions were tracked and recorded. All authors extracted data from 3 articles to pilot-test the spreadsheet. The research team discussed any ambiguity, resolved differences in interpretation, and modified the data extraction spreadsheet. Subsequently, each article underwent independent data extraction by YZ and another author (6 trained reviewers). The research team met throughout the study period every other week to resolve any discrepancies. A total of 15 original study authors were contacted to request missing information (eg, mean, SD, and sample size), and no additional data were received.

### Assessment of Methodological Quality

The reviewers assessed the included studies for methodological rigor using standardized critical appraisal instruments from the 13-item Joanna Briggs Institute (JBI) Critical Appraisal Checklist for RCT and the 9-item JBI Critical Appraisal Checklist for quasi-experimental studies [29]. Reviewers answered each risk of bias item as “yes” (score=1), “no” (score=0), “unclear” (score=0), or “not applicable.” Possible composite scores ranged from 0 to 9 for quasi-experimental studies and 0-13 for RCTs, with higher scores indicating less risk of bias and better study quality. The applicable score (range 0-1) was calculated by dividing the composite score by the maximum score possible after subtracting any “not applicable” responses [30]. All studies were double-coded, and any disagreements were resolved through discussion with the research team [26].

### Data Synthesis and Meta-Analysis

#### Data Synthesis

Data synthesis was completed on all articles that met the inclusion criteria. Only primary study results were included if multiple articles were published from the same intervention study. Simple descriptive statistics (ie, mean, SD, frequency, and percentage) were used to summarize study characteristics (eg, study design and participant characteristics) and key features of interventions (ie, theory, mode of delivery, number of sessions, frequency, and total length). Intervention content was grouped and narratively summarized according to the description of the intervention components.

#### Meta-Analytical Procedure

An a priori decision was made to only include studies in the meta-analysis if at least 2 studies used the same instrument to assess the same psychosocial outcome [31]. Standardized mean differences (ie, Hedges *g*) were calculated to compare intervention effectiveness across studies that used different scales or measurements. Mean differences between the scores before the intervention and the follow-up assessment after the intervention were calculated for pre-post interventions. Similarly, for the RCT studies, the results from follow-up in each study were selected and analyzed using difference scores from before and after the intervention for both intervention and control groups, with the pooled SDs. We computed the overall effect size across different time points for studies with multiple follow-ups. By doing so, we captured the time-varying effect on intervention effectiveness [31]. The overall effect (including all information across all time points) and time-varying effects, including the interim effect (during the intervention period), immediate effect (after the intervention), short-term effect (follow-up ≤8 weeks after completion of the intervention), and long-term effect (follow-up >8 weeks after completion of the intervention), were calculated. A cutoff of 8 weeks was chosen because it was the median length of the follow-up period across the included studies.

To assess study heterogeneity, the *I²* statistic was examined. The *I²* statistic quantifies the proportion of total variance across studies caused by a fundamental difference between trials rather than chance. An *I²* statistic of <25% indicates low heterogeneity, between 25% and 75% indicates moderate heterogeneity, and >75% indicates high heterogeneity [32]. Lower heterogeneity is better. Funnel plots (ie, to visually assess the asymmetry) and Egger test (ie, to test the asymmetry statistically) assessed publication bias [33]. In funnel plots, if points are distributed equally between positive and negative effects, bias is lacking; variability is expected to be greater near the bottom of the chart among smaller sample size studies. For the analysis of data from studies with more than 1 digital psychosocial intervention group, we compared each digital psychosocial intervention group to the control group separately. Additionally, subgroup analysis was planned based on the review’s focus on examining the effect of delivery mode, type of control condition, and dosage on outcomes. Furthermore, we performed sensitivity analyses by including and excluding studies with extreme weights in the analyses. We used the DerSimonial-Laird random-effects model to weight and pool the individual estimates to capture variance across different studies, as all included studies were conducted in heterogeneous populations across various settings [34]. We performed all statistical and meta-analyses using STATA (version 17; StataCorp LLC).

## Results

### Search Results

**Figure 1:**
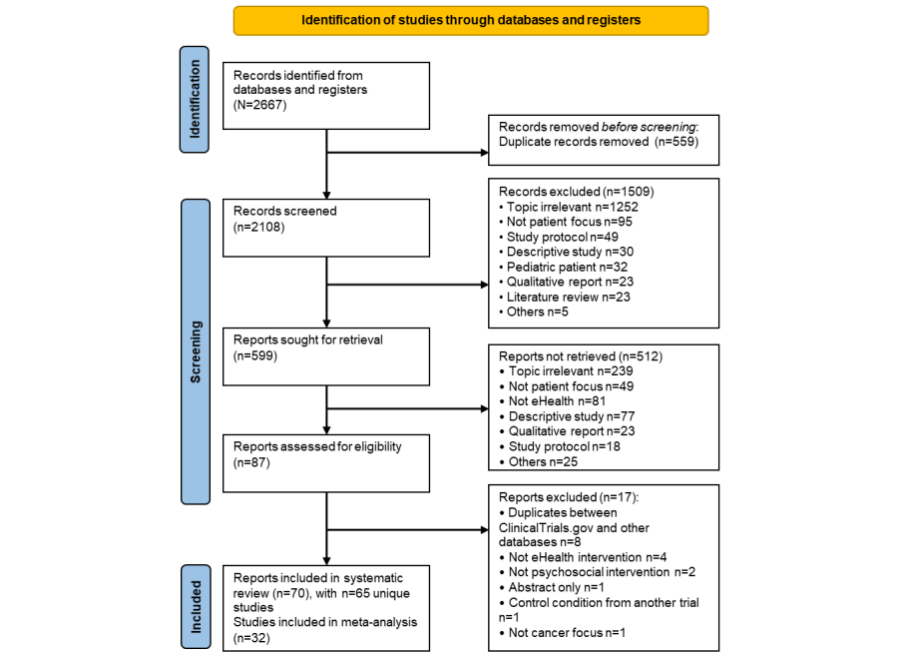
PRISMA (Preferred Reporting Items for Systematic Reviews and Meta-Analyses) flow diagram.

After removing duplicates, a total of 2108 studies were identified. Figure 1 shows a flow diagram of studies identified, screened, included, and excluded from this systematic review and meta-analysis. After screening titles and abstracts and applying inclusion and exclusion criteria, a total of 70 records with 65 unique studies (for multiple manuscripts published from the same intervention study, only primary manuscripts were included) were included in the systematic review [35-99] and 32 studies [35,36,38,40,41,43,44,47,50,53-55,57,64,66,68-72,​^74^,^77^,^80^,^82^,^83^,^86^,^88^,^91^,^93^,^95^,^96^,^99^] with available data were included in the meta-analysis. A total of 33 studies were excluded from the meta-analysis because either data were unavailable to calculate the effect size (n=14) [42,46,51,62,73,76,78,79,81,84,87,89,94,98] or no other study used the same measure (n=19) [37,39,45,48,49,52,​^56^,^58^-^61^,^63^,^65^,^67^,^75^,^85^,^90^,^92^,^97^].

### Study Characteristics

#### Overview

Of the 65 studies, 48 (74%) were RCTs [35-37,​^39^-^50^,^52^-^57^,^59^-^65^,^72^,^73^,^76^-^82^,^85^,^89^-^91^,^93^-^99^], and 17 (26%) were quasi-experimental [38,51,58,​^66^-^71^,^74^,^75^,^83^,^84^,^86^-^88^,^92^]. More than half (n=37, 58%) of the studies were conducted in the United States [36-40,44,48-50,54,56-61,63,​^65^,^67^-^69^,^71^,^72^,^74^,^77^,^79^,^80^,^83^-^85^,^87^-^90^,^94^,^97^,^98^], and the rest were from the Netherlands (n=9, 14%) [35,41,43,47,64,70,91,92,96], Australia (n=5, 8%) [45,66,75,86,95], and other countries (eg, Denmark and Ireland). In total, 10,361 patients (mean 159, SD 166; range 9-803 patients per study) were included: 7098 female patients and 3263 male patients; 1045 caregivers or partners were enrolled (mean 16, SD 54; range 9-244 caregivers or partners per study), including 781 female individuals and 264 male individuals. The average age of patients ranged from 39.9 to 72 years, and the average age of caregivers or partners ranged from 51.5 to 58.8 years. In the 33 studies that provided information about race and ethnicity, most patients (n=3495, 90%) and family members (n=259, 97%) were described as “White” or “Caucasian.” The cancer diagnoses varied across studies, with the most prevalent being breast cancer (n=24, 37%) [35,37,38,42,​^44^,^52^,^54^,^58^,^60^,^61^,^64^-^66^,^70^-^72^,^74^,^76^,^80^,^82^,^84^,^91^,^93^,^95^], mixed cancer diagnosis (n=19, 29%) [36,40,45,​^47^,^50^,^51^,^53^,^57^,^62^,^69^,^75^,^77^,^78^,^81^,^83^,^94^,^96^,^97^,^99^], and prostate cancer (n=7, 11%) [39,48,56,67,85,88,98]. The attrition rate ranged from 0% to 76%, with a median of 16.8% (mean 20.4%, SD 13.7%). The recruitment rate ranged from 4.4% to 94.2%, with a median of 59.5% (mean 56%, SD 24.6%). Detailed information about the study and sample characteristics from the included studies is provided in Table S1 in Multimedia Appendix 2 [35-99].

#### Control Condition

Of the 48 RCTs, 29 (60%) studies included a usual care control group [35,36,39,41-44,46,47,52,54,55,62,64,65,73,76-80,82,​^85^,^91^,^93^-^96^,^99^], and 19 (40%) included an active control [37,40,45,48-50,53,56,57,59-61,63,72,81,89,90,97,98]. Among the 17 quasi-experimental studies, 11 (65%) did not have a control group [38,51,67-71,74,86-88], and 6 (35%) studies included a usual care control group [58,66,75,83,84,92].

#### Outcome Assessment

A total of 21 studies had 1 follow-up assessment [22,38,45,47,48,50,51,57,68-70,72,74,76-78,88-90,98,99], 23 had 2 follow-up assessments [35,39,44,46,52-55,​^63^-^65^,^67^,^71^,^75^,^79^,^83^,^84^,^86^,^87^,^92^,^93^,^95^,^97^], 11 had 3 follow-up assessments [37,40-43,60-62,66,80,91], and 7 had 4 or more follow-up assessments [49,56,58,59,73,81,85]. The timing of follow-up assessments varied, ranging from immediately to 6 months after the intervention. The commonly reported outcomes and relevant measures are reported below in *Aim 2: Effects on Patients’ and Family Members’ Psychosocial Outcomes*.

#### Quality Assessment (Risk of Bias)

The quality assessment scores of the included studies are summarized in Table S1 in Multimedia Appendix 2. Overall, the RCT studies’ general quality was mixed, with applicable scores ranging from 0.38 to 0.91 (mean 0.61, SD 0.12). Quasi-experimental studies were generally of moderate to high quality, with applicable scores ranging from 0.63 to 0.89 (mean 0.75, SD 0.08) on the JBI Critical Appraisal Checklist for quasi-experimental studies. The publication year and applicable appraisal score were not significantly correlated in RCTs (*r*=0.12; *P*=.40) and quasi-experimental studies (*r*=–0.04; *P*=.88).

### Aim 1: Intervention Characteristics

#### Overview

There was large heterogeneity in intervention components, theoretical or conceptual framework, type of intervention (ie, tailored or standardized), mode of delivery, prescribed dosage (ie, number of sessions, frequency, and length), and received dosage (Table S2 in Multimedia Appendix 2).

#### Intervention Components

A total of 37 (57%) out of 65 studies included a single intervention component [36,38-40,44,45,47,51,52,55-57,63-66,​^68^,^69^,^71^-^77^,^79^,^80^,^82^-^84^,^86^,^87^,^90^,^91^,^93^,^94^,^98^], 13 (20%) studies included 2 intervention components [35,41,43,46,48,50,62,67,78,85,88,95,96], and 15 (23%) studies included 3-5 intervention components [37,42,​^49^,^53^,^54^,^58^-^61^,^70^,^81^,^89^,^92^,^97^,^99^]. The most common intervention components were information and resources, or psychoeducation (n=29, 45%) [35,37,39-43,46,48,49,​^52^-^56^,^58^-^61^,^70^,^81^,^82^,^87^-^90^,^92^,^95^,^97^], and cognitive-behavioral strategies (n=20, 31%) [44,45,47,50,54,57,63,64,​^67^,^68^,^71^,^74^,^75^,^80^,^85^-^87^,^89^,^91^,^98^].

#### Theoretical or Conceptual Framework

More than half (n=38, 59%) of the included studies did not identify a conceptual or theoretical framework [37,41,42,45-47,51,52,54,55,57-63,66,69,71,73,75,76,78-81,85-90,92,93,95,97,98].

#### Standardized or Tailored Intervention

Of the 65 studies, 26 (40%) included both standardized and tailored interventions [37,39,42,43,46,47,49,53,55,​^59^-^61^,^64^,^68^,^70^,^73^-^75^,^77^-^79^,^81^,^85^,^89^,^92^,^94^], 28 (43%) studies included only standardized interventions [36,38,​^41^,^44^,^50^,^51^,^54^,^56^-^58^,^63^,^65^-^67^,^69^,^71^,^72^,^76^,^80^,^82^-^84^,^87^,^88^,^91^,^93^,^95^,^98^], and 11 (17%) studies had only tailored interventions [35,40,45,48,52,62,86,90,96,97,99].

#### Modes of Delivery

The majority of studies conducted interventions through an internet website (n=40, 62%) [35-37,39-50,​^53^,^55^,^59^-^62^,^64^,^67^,^68^,^70^,^72^,^75^,^77^,^81^,^82^,^85^,^88^-^91^,^93^,^95^-^99^] or smart device app (n=8) [43,52,56,57,63,69,80,87]. A total of 7 (11%) studies conducted interventions through virtual reality [51,66,73,76,78,83,84], 3 (5%) studies through telehealth [54,74,79], and 2 (3%) studies through a computer program [38,65]. Electronic health information systems [92], interaction portals [58], and videoconferences [86] were each used in 1 study. Overall, 2 studies used multimodal interventions delivered through the combination of either telephone and videoconference [94] or internet and telephone [61].

#### Dosage

The dosage prescribed and received were highly variable. The number of intervention sessions ranged from 1 to 56, with a median of 6. A total of 27 (42%) studies did not specify the prescribed dose; 19 (29%) only stated the number of days participants had access to the intervention [35,36,42,49,56,59-62,67,70,80,81,85,87,89,92,95,99] and 8 (12%) did not provide information on the prescribed dosage [37,39,40,52,55,58,72,73]. Frequency was highly variable, with self-paced (n=26, 40%) as the most common [35,36,42,45,49,50,56,59-63,67,70,80,81,85,87-90,92,95-97,99], meaning no specific intervention frequency was defined and the intervention content was available throughout the study period. The other common frequencies of intervention sessions were weekly (n=17, 26%) [38,41,44,45,47,53,54,64,​^68^,^71^,^74^,^75^,^77^,^86^,^91^,^94^,^98^] and 1-time intervention sessions (n=8, 12%) [48,65,66,76,78,82-84]. The median length of the intervention was 8 weeks, with the length ranging from 1 hour (ie, use of the intervention on an iPad for an hour) to 24 months. Received dosage was defined as the uptake of the intervention by the participants. A total of 18 (28%) studies did not report the received dosage [36,37,39,48,52,56,58,65,69,75,​^76^,^78^,^79^,^82^,^85^,^86^,^93^,^94^]. Various information was reported, including attendance rate, number of times participants used the app, frequency with which participants logged into the website, number of website pages reviewed, skill practice time, and intervention session completion rate. Most of the interventions (n=43, 66%) were self-delivered without an interventionist, with self-paced being most common [35-37,39,40,42,44,45,48-53,55,57-60,62,63,67,69-73,​^75^,^76^,^78^,^80^-^84^,^87^,^88^,^91^-^93^,^95^,^96^,^99^].

### Aim 2: Effects on Patients’ and Family Members’ Psychosocial Outcomes

#### Patients’ Outcomes

##### Overview

A meta-analysis was conducted on 32 studies. Overall, 5 outcomes were examined. A summary of the interventions’ overall effect sizes; time-varying effect sizes for quality of life, anxiety, depression, distress, and self-efficacy; and heterogeneity statistics for each outcome is displayed in Table 1. The forest plots for overall effect sizes and time-varying effects are displayed in Multimedia Appendix 3. The funnel plots for overall effect sizes and time-varying effects are displayed in Multimedia Appendix 4.

**Table 1:** Summary of the meta-analysis.

Population, outcome, measure, and value					Effect at different time points								
					Overall^a^		Immediate		Interim		Short		Medium
**Patient**													
	**QOL^b^**												
		**FACT-B^c^**											
			Pooled ES^d^, Hedges *g* (95% CI)	0.13 (–0.05 to 0.31)		—^e^		—		—		—	
			*I* ^2^	62.3		—		—		—		—	
			Heterogeneity, *χ*^2^ (*df*)	10.61 (4)		—		—		—		—	
			*P* value	.03		—		—		—		—	
		**FACT-G^f^**											
			Pooled ES, Hedges *g* (95% CI)	–0.04 (–0.17 to 0.09)		—		—		—		—	
			*I* ^2^	0		—		—		—		—	
			Heterogeneity, *χ*^2^ (*df*)	1.91 (4)		—		—		—		—	
			*P* value	.43		—		—		—		—	
		**QLQ-30^g^**											
			Pooled ES, Hedges *g* (95% CI)	0.05 (–0.04 to 0.14)		—		—		—		—	
			*I* ^2^	58.4		—		—		—		—	
			Heterogeneity, *χ*^2^ (*df*)	19.95 (6)		—		—		—		—	
			*P* value	.03		—		—		—		—	
		**SF36^h^**											
			Pooled ES, Hedges *g* (95% CI)	0.03 (–0.10 to 0.15)		—		—		—		—	
			*I* ^2^	14.4		—		—		—		—	
			Heterogeneity, *χ*^2^ (*df*)	8.41 (8)		—		—		—		—	
			*P* value	.31		—		—		—		—	
		**Overall**											
			Pooled ES, Hedges *g* (95% CI)	0.05 (–0.01 to 0.10)		0.95 (–1.99 to 3.89)		–0.16 (–0.39 to 0.06)		2.25 (0.36 to 4.14)		0.18 (0 to 0.35)	
			*I* ^2^	42.7		100		70		98		18.3	
			Heterogeneity, *χ*^2^ (*df*)	48.12 (20)		93227.62 (19)		3.34 (1)		203.50 (4)		7.35 (6)	
			*P* value	.01		<.001		.07		<.001		.29	
	**Anxiety and depression**												
		**HADS^i^ total score**											
			Pooled ES, Hedges *g* (95% CI)	–0.72 (–1.89 to 0.46)		–0.04 (–0.23 to 0.16)		—		–0.22 (–0.54 to 0.10)		0.14 (–0.09 to 0.38)	
			*I* ^2^	97.6		0		—		0		0	
			Heterogeneity, *χ*^2^ (*df*)	165.82 (14)		3.71 (4)		—		0.19 (1)		0.51 (1)	
			*P* value	<.001		.45		—		.66		.47	
	**Depression**												
		**HADS-depression**											
			Pooled ES, Hedges *g* (95% CI)	–0.13 (–0.23 to –0.02)		—		—		—		—	
			*I* ^2^	0		—		—		—		—	
			Heterogeneity, *χ*^2^ (*df*)	4.17 (7)		—		—		—		—	
			*P* value	.73		—		—		—		—	
		**CESD^j^**											
			Pooled ES, Hedges *g* (95% CI)	0.10 (–0.10 to 0.30)		—		—		—		—	
			*I* ^2^	0		—		—		—		—	
			Heterogeneity, *χ*^2^ (*df*)	0.99 (4)		—		—		—		—	
			*P* value	.91		—		—		—		—	
		**PHQ9^k^**											
			Pooled ES, Hedges *g* (95% CI)	–0.05 (–0.17 to 0.08)		—		—		—		—	
			*I* ^2^	0		—		—		—		—	
			Heterogeneity, *χ*^2^ (*df*)	0.78 (1)		—		—		—		—	
			*P* value	.38		—		—		—		—	
		**Multiple scales**											
			Pooled ES, Hedges *g* (95% CI)	0.32 (–0.35 to 0.99)		—		—		—		—	
			*I* ^2^	95		—		—		—		—	
			Heterogeneity, *χ*^2^ (*df*)	19.86 (1)		—		—		—		—	
			*P* value	<.001		—		—		—		—	
		**Overall**											
			Pooled ES, Hedges *g* (95% CI)	0.03 (–0.10 to 0.16)		0.06 (–0.10, 0.22)		-0.04 (–0.22, 0.14)		—		—	
			*I* ^2^	60.9		69.4		29.8		—		—	
			Heterogeneity, *χ*^2^ (*df*)	40.77 (16)		58.85 (16)		4.27 (1)		—		—	
			*P* value	<.001		<.001		0.23		—		—	
	**Anxiety**												
		**HADS-anxiety**											
			Pooled ES, Hedges *g* (95% CI)	0.32 (–0.20 to 0.84)		—		—		—		—	
			*I* ^2^	94.3		—		—		—		—	
			Heterogeneity, *χ*^2^ (*df*)	123.33 (7)		—		—		—		—	
			*P* value	<.001		—		—		—		—	
		**SATI^l^**											
			Pooled ES, Hedges *g* (95% CI)	–0.19 (–0.41 to 0.04)		—		—		—		—	
			*I* ^2^	26.8		—		—		—		—	
			Heterogeneity, *χ*^2^ (*df*)	5.46 (4)		—		—		—		—	
			*P* value	.24		—		—		—		—	
		**Overall**											
			Pooled ES, Hedges *g* (95% CI)	0.12 (–0.19 to 0.43)		–0.10 (–0.19 to 0)		–0.04 (–0.19 to 0.12)		–0.13 (–0.43 to 0.17)		—	
			*I* ^2^	90.2		6.7		35.1		10.5		—	
			Heterogeneity, *χ*^2^ (*df*)	132.99 (13)		13.94 (13)		6.16 (4)		1.12 (1)		—	
			*P* value	<.001		.38		.19		.29		—	
	**Distress**												
		**DT^m^**											
			Pooled ES, Hedges *g* (95% CI)	0.98 (–0.18 to 2.14)		0.51 (0.10 to 0.92)		—		—		—	
			*I* ^2^	98.5		54.2		—		—		—	
			Heterogeneity, *χ*^2^ (*df*)	332.71 (2)		4.37 (2)		—		—		—	
			*P* value	<.001		.11		—		—		—	
	**Self-efficacy**												
		**CBI^n^**											
			Pooled ES, Hedges *g* (95% CI)	–1.41 (–4.02 to 1.20)		2.56 (–1.22 to 6.35)		—		—		—	
			*I* ^2^	99		98.2		—		—		—	
			Heterogeneity, *χ*^2^ (*df*)	1.06 (1)		55.43 (1)		—		—		—	
			*P* value	.29		<.001		—		—		—	
**Family member**													
	**Depression**												
		**HADS-depression**											
			Pooled ES, Hedges *g* (95% CI)	–0.25 (–0.72 to 0.21)		—		—		—		—	
			*I* ^2^	0		—		—		—		—	
			Heterogeneity, *χ*^2^ (*df*)	0.41 (1)		—		—		—		—	
			*P* value	.52		—		—		—		—	
	**Anxiety**												
		**HADS-anxiety**											
			Pooled ES, Hedges *g* (95% CI)	–0.23 (–0.70 to 0.23)		—		—		—		—	
			*I* ^2^	0		—		—		—		—	
			Heterogeneity, *χ*^2^ (*df*)	0.65 (1)		—		—		—		—	
			*P* value	.42		—		—		—		—	

^a^The overall effect accounts for time-varying effect across different time points.

^b^QOL: quality of life.

^c^FACT-B: Functional Assessment of Cancer Therapy–Breast.

^d^ES: effect size.

^e^Not applicable.

^f^FACT-G: Functional Assessment of Cancer Therapy–General.

^g^QLQ-30: Quality of Life Questionnaire, 30 items.

^h^SF36: Short Form Survey 36-item.

^i^HADS: Hospital Anxiety and Depression Scale.

^j^CESD: Center for Epidemiologic Studies Depression Scale.

^k^PHQ9: Patient Health Questionnaire-9.

^l^SATI: State-Trait Anxiety Inventory.

^m^DT: Distress Thermometer.

^n^CBI: Coping Behaviors Inventory.

##### Quality of Life

Quality of life was measured by the Functional Assessment of Cancer Therapy–Breast [44,54,80,93,95], Functional Assessment of Cancer Therapy–General [38,53,57,86,88], European Organization for Research and Treatment of Cancer Quality of Life Questionnaire, 30-items [35,40,43,74,91,96,99], and 36-item Short Form Survey [41,54,64,70,71]. Overall, a total of 21 studies with 1847 participants in the intervention groups showed an increase in quality of life, with a mean difference between groups of Hedges *g*=0.05 (95% CI –0.01 to 0.10). The impact of heterogeneity within the studies was significant (*I*^2^=42.7%; *P*=.01). With respect to publication bias, the funnel plot displayed a greater number of studies toward the top of the mean (Egger test, *P*<.001). The time-varying effects were as follows: Hedges *g*=–0.16 (95% CI –0.39 to 0.06) for the interim effect; Hedges *g*=0.95 (95% CI –1.99 to 3.89) for the immediate effect; Hedges *g*=2.25 (95% CI 0.36-4.14) for the short-term effect; and Hedges *g*=0.18 (95% CI 0-0.35) for the long-term effect. The statistical heterogeneity among studies was *I*^2^=70% (*P*=.07) for the interim effect; *I*^2^=100% (*P*<.001) for the immediate effect; *I*^2^=98% (*P*<.001) for the short-term effect; and *I*^2^=18.3% (*P*=.29) for the long-term effect.

##### Anxiety and Depression

Hospital Anxiety and Depression Scale (HADS) total scores (without subscale scores reported) were reported in 5 studies with 338 participants in the intervention groups [43,47,64,86,91]. Overall, participants receiving interventions reported decreased anxiety and depression with a standardized mean difference of Hedges *g*=–0.72 (95% CI –1.89 to 0.46). The heterogeneity within the studies was significant (*I*^2^=97.6%; *P*<.001). The funnel plot was found to be asymmetric, and Egger test was found to be not statistically significant (*P*=.77). The time-varying effects were as follows: Hedges *g*=–0.04 (95% CI –0.23 to 0.16) for the immediate effect; Hedges *g*=–0.22 (95% CI –0.54 to 0.10) for the short-term effect; and Hedges *g*=0.14 (95% CI –0.09 to 0.38) for the medium-term effect. The statistical heterogeneity among studies was *I*^2^=0% across all time-varying effects.

##### Depression

Depression was assessed by the HADS-depression subscale [50,69,74,86,95,96,99], Center for Epidemiologic Studies Depression Scale [41,68,71,77], Patient Health Questionnaire-9 (PHQ-9) [40,53], and a combination of the PHQ-9 and HADS-anxiety [43,57] in 1509 participants in the intervention groups. Overall, interventions were not more effective than control conditions for reducing depression (Hedges *g*=0.03, 95% CI –0.10 to 0.16), with a high heterogeneity of 60.9% (*P*<.001). With respect to publication bias, the funnel plot displayed a greater number of studies toward the top of the mean (Egger test, *P*=.25). The time-varying effects were as follows: Hedges *g*=0.06 (95% CI –0.10 to 0.22) for the immediate effect and Hedges *g*=–0.04 (95% CI –0.22 to 0.14) for the interim effect. The statistical heterogeneity among studies was *I*^2^=69.4% for the immediate effect and *I*^2^=29.8% for the interim effect.

##### Anxiety

Anxiety was assessed by the HADS-anxiety subscale [57,64,69,74,86,95,96,99], State-Trait Anxiety Inventory (STAI) [66,71,72,82,83], and a combination of the STAI and HADS-anxiety [43] in 1075 participants in the intervention groups. Overall, interventions were not more effective than control conditions for reducing anxiety (Hedges *g*=0.12, 95% CI –0.19 to 0.43), with high heterogeneity of 90.2% (*P*<.001). The funnel plot displayed a greater number of studies toward the top of the mean (Egger test, *P*=.46). The interim effect was Hedges *g*=–0.04 (95% CI –0.19 to 0.12), and the immediate effect was Hedges *g*=–0.10 (95% CI –0.19 to 0), and the short-term effect was Hedges *g*=–0.13 (95% CI –0.43 to 0.17). The statistical heterogeneity among studies was *I*^2^=35.1% for the interim effect, *I*^2^=6.7% for the immediate effect, and *I*^2^=10.5% for the short-term effect.

##### Distress

Psychological distress was assessed in 182 participants in the intervention groups using the distress thermometer [35,69,91]. Overall, participants in the intervention groups showed no reduction in distress, with a mean difference between groups of Hedges *g*=0.98 (95% CI –0.18 to 2.14). The impact of heterogeneity within the studies was significant (*I*^2^=98.5%; *P*<.001). Regarding publication bias, the funnel plot displayed a symmetric distribution around the mean effect (Egger test, *P*=.46). The immediate effect was Hedges *g*=0.51 (95% CI 0.10-0.92), with statistical heterogeneity *I*^2^=54.2%.

##### Self-Efficacy

Self-efficacy was measured by the Coping Behaviors Inventory in 174 participants in the intervention groups [44,55]. Overall, participants in the intervention groups did not report improvement in self-efficacy, with a standardized mean difference of Hedges *g*=–1.41 (95% CI –4.02 to 1.20). However, the impact of heterogeneity within studies was significant (*I*^2^=99%; *P*<.001). Regarding the publication bias, the funnel plot displayed a symmetric distribution around the mean effect (Egger test, *P*=.22). The immediate effect was Hedges *g*=2.56 (95% CI –1.22 to 6.35) with high heterogeneity (*I*^2^=98.2%; *P*<.001).

#### Subgroup Analyses

Given the heterogeneity of reporting on dosage information and limited data, the subgroup analysis of dosage on intervention effect was not conducted. Table 2 includes the results of the subgroup analysis on the effect on quality of life, depression and anxiety, and distress. Overall, the associations between delivery mode and control condition with patient outcomes were not statistically significant (*P*>.05).

**Table 2:** Subgroup analyses on the effect of delivery mode (internet vs noninternet) and control condition (usual care vs active control) on patient outcomes.

Outcome and moderators			Effect size, Hedges *g* (95% CI)		SE	*P* value
**Quality of life (27 studies)**						
	Delivery mode	0.04 (–0.06 to 0.14)		0.05		.45
	Control condition	–0.01 (–0.99 to 0.06)		0.04		.78
**HADS^a^ total (6 studies)**						
	Delivery mode	0.16 (–0.30 to 0.62)		0.24		.50
	Control condition	N/A^b^		N/A		N/A
**Depression (21 studies)**						
	Delivery mode	0.10 (–0.09 to 0.29)		0.10		.31
	Control condition	–0.04 (–0.17 to 0.09)		0.07		.55
**Anxiety (15 studies)**						
	Delivery mode	0.01 (–0.07 to 0.09)		0.04		.79
	Control condition	–0.06 (–0.34 to 0.22)		0.14		.67
**Distress (8 studies)**						
	Delivery mode	N/A		N/A		N/A
	Control condition	–0.03 (–0.34 to 0.28)		0.15		.86

^a^HADS: Hospital Anxiety and Depression Scale.

^b^N/A: not applicable.

#### Family Members’ Outcomes

The forest plots for overall effect sizes are displayed in Multimedia Appendix 5. The funnel plots for overall effect sizes are displayed in Multimedia Appendix 6. For family members’ data, we pooled 2 studies [36,69] on anxiety and depression for the meta-analysis with 68 participants in the intervention groups. Due to the small sample size, no time-varying effect or subgroup analysis was calculated. The overall effect on anxiety was Hedges *g*=–0.23 (95% CI –0.70 to 0.23), with heterogeneity of *I*^2^=0% (*P*=.42). The overall effect on depression was Hedges *g*=–0.25 (95% CI –0.72 to 0.21), with heterogeneity of *I*^2^=0% (*P*=.52). Regarding publication bias, the funnel plot displayed asymmetrical scattered points with statistical significance (Egger test, *P*<.001).

## Discussion

### Overview

This systematic review and meta-analysis of 65 unique digital psychosocial intervention studies for patients with cancer and their family members provides strong evidence that psychosocial interventions delivered through digital health significantly improve psychosocial outcomes. There were 3 major findings. First, this review included a large group of participants with various cancer diagnoses; however, underrepresented populations affected by cancer were not included, and the results predominantly focused on White patients. Second, we found that various intervention modes and components were used. There is a lack of specificity with respect to the description of interventions or theoretical basis for interventions, which may hinder future replication or refinement of the interventions and understanding of underlying mechanisms. Third, despite high heterogeneity across studies, the available data suggest that digital psychosocial interventions effectively improve some psychosocial outcomes, including patients’ quality of life, anxiety, and depression.

### Principal Findings

First, the majority of participants in the included studies were White and female, which does not reflect the broader patient population with cancer, including non-White ethno-racial groups (ie, African American or Black, American Indian and Alaska Native, Asian, Native Hawaiian or other Pacific Islander, and Hispanic or Latino populations). It is well documented in the literature that the impact of cancer on psychological distress and quality of life is worse for racial and ethnic minority groups [100-102]. Therefore, future trials should include more participants from underrepresented groups to reduce health care disparities and improve generalizability in diverse populations [103]. Family members and caregivers were rarely included in the studies reviewed. However, there is ample evidence that family members and caregivers experience significant caregiver burden, worsening quality of life, and difficulty with psychological adjustment, therefore needing support [104,105]. Previous systematic reviews suggest that interventions targeting problem-solving and communication skills may ease the burden related to patient care and improve caregivers’ quality of life [106]. Many reviews focus on the evaluation of nondigital interventions targeting the psychosocial experience in family members and caregivers, including several reviews of caregiver interventions [9,107-109]. Therefore, with growing technology usage, more digital interventions are needed to address family members’ or caregivers’ needs.

Few RCTs met all quality criteria, including blinding, analysis by treatment assignment, and standardized outcome assessment [110]. While concealing assignments from participants and those delivering interventions is not always possible, single blinding of assessors should occur in well-designed research. Few studies used power calculations for sample size, making it difficult to determine whether sample sizes were adequate [111]. Generally, results from group sizes <20 are questionable. There are several effective strategies known to increase the retention rate, such as adding monetary incentives and using an open trial design [112,113]. The critical appraisal also depends on comprehensive reporting of study details, which were limited in the identified studies. Although attempts have been made to improve reporting using the CONSORT (Consolidated Standards of Reporting Trials) statement for RCTs and the Transparent Reporting of Evaluations with Nonrandomized Designs (TREND) statement for nonrandomized intervention studies in the early 2000s [114,115], we did not see improvement in appraisal scores over time. The main limitations of the results include underpowered and methodologically weaker studies. These highlight the need for improved methodologies in future research, as the overall methodological quality was moderate.

Second, this study identified various intervention modes and components, which is consistent with a previous systematic review of psychosocial interventions for patients with advanced cancer, which identified similar intervention components, including psychoeducation and CBT-based intervention, as 2 of the most common [11]. However, more than half of the included studies did not use theoretical or conceptual frameworks to guide the development of intervention components or selection of outcomes. The lack of a theoretical framework leads to a lack of clarity about the mechanisms through which intervention components impact psychosocial outcomes [116]. In future research, theories or conceptual frameworks need to be incorporated to help us better understand the mechanisms that explain the changes in psychosocial outcomes when using digital health interventions.

In addition, the prescribed dosage information (ie, the number of sessions, duration, and frequency) was inconsistently reported, making it difficult to estimate an efficacious intervention dose. Most of the interventions were self-paced, without the involvement of an interventionist, which gives the patient autonomy to choose which intervention component or module they would like to focus on and how much time to allocate. However, there is a lack of information on intervention uptake, which may have influenced the effectiveness of interventions. Approaches that tackle barriers to adherence at various levels (eg, individual, family, clinician, agency, and environment) and improve engagement should be implemented [117]. For example, a scoping review about engagement strategies in digital interventions for mental health promotion recommended personalized feedback, e-coaching to guide content and individual progress, social platforms and interaction with peers, content gamification, reminders, and ease of use [118].

Third, we found some significant improvement in the patient’s quality of life. Some studies with a smaller number of participants or with a focus on internet-based interventions reported an improvement, but the results from these studies were not consistent [21,119]. This meta-analysis, including 21 studies, revealed a small effect size for overall effectiveness of digital health interventions in improving patients’ quality of life (Hedges *g*=0.05, 95% CI –0.01 to 0.10), with time-varying effects shown as promising. Another meta-analysis that pooled 16 studies demonstrated a larger positive effect of mHealth interventions on the quality of life of patients with cancer (standardized mean difference 0.28, 95% CI 0.03-0.53) [21]. Another meta-analysis that included 6 internet-based psychoeducational interventions for patients with cancer showed no significant improvement in quality of life (mean difference 1.10, 95% CI –4.42 to 6.63) [119]. Importantly, our analyses found the largest improvements in quality of life occurred from post intervention to 8 weeks (Hedges *g*=2.25, 95% CI 0.36-4.14). The effect of psychosocial interventions decreased after 8 weeks of follow-up, suggesting that interventions may need booster sessions or tailoring to time-sensitive needs in order to maintain effectiveness in the long term. This result was limited by substantial inconsistency across studies in all evaluation periods except the medium-term effect [32]. In addition, given the heterogeneity of follow-up periods in selected studies, the time-varying effect was only tested with a small number of studies, not in the 21 studies we used to calculate the overall effect.

This meta-analysis was able to demonstrate the effectiveness of digital health interventions on both anxiety and depression (measured by HADS: Hedges *g*=–0.72, 95% CI –1.89 to 0.46). Our finding was partially consistent with the other meta-analyses. One study showed that internet-based psychoeducational interventions had a significant effect on decreasing depression (standardized mean difference −0.58, 95% CI −1.12 to −0.03), but found no evidence for effects on distress (standardized mean difference −1.03, 95% CI −2.63 to 0.57) [119]. However, there was considerable heterogeneity in measurements among the studies included in the review by Wang et al [119]; it is difficult to determine how meaningful it is to make direct comparisons between the studies included in this meta-analysis and past reports. Possible ways to address this problem could be using similar outcome measures and a standardized study report.

Our meta-analysis demonstrated that the interventions were effective in reducing anxiety and depression in family caregivers. However, the effect size was small, perhaps due to the limited number of studies. This is partially consistent with findings from another meta-analysis, which found depressive symptoms decreased from baseline to post intervention (Hedges *g*=–0.44, 95% CI –1.03 to 0.15) [120], while anxiety remained relatively stable when comparing intervention to control either at postintervention (Hedges *g*=0.12, 95% CI –0.16 to 0.44) or during follow-up (Hedges *g*=–0.08, 95% CI –0.34 to 0.19).

### Strengths and Limitations

This review’s strength lies in its rigorous design, sophisticated data synthesis, and enduring empirical contributions. We acknowledge that our literature search was conducted 4 years before manuscript submission. The findings and contributions from our research remain pertinent and enduring. This is because, as digital psychosocial interventions continue to evolve, the core intervention content and outcomes have remained relatively consistent over the past 4 years. It would be valuable to conduct a reassessment of the evolving body of evidence concerning digital psychosocial interventions that have emerged since the onset of the COVID-19 pandemic. Moreover, this study was conducted in line with best practices by double-coding and following the PRISMA guidelines. The meta-analysis, including subgroup analysis, was conducted using appropriate methods for combining studies across various follow-up periods. Although we did an extensive search at the start of this review, we may have missed some critical studies, unreported, or unfinished studies. If all data were available, the meta-analysis could have reduced the chances of inflated type-1 error for both observed and unobserved effects that were available for assessment [121]. There was not enough data to perform post hoc analyses to examine the effect of factors such as intervention components and length of intervention on outcomes due to insufficient data.

### Conclusions

Patients with cancer and their family members need high-quality psychosocial interventions throughout the cancer trajectory. Digital technologies provide a platform to deliver evidence-based psychosocial interventions from a distance, without the heightened risk of contracting viruses, especially for patients with cancer whose immune systems are compromised. This study comprehensively synthesized the effects of digital psychosocial interventions for people affected by cancer. Our findings suggest that digital health interventions are effective for adult patients with cancer and their family members. Further research development in this area needs to include large, high-quality studies with a clear description of the methodology, theoretical foundations, and standardized tools to permit inclusion in meta-analyses to inform the effectiveness of interventions for a better understanding of the mechanisms.

## Appendix

Multimedia Appendix 1 Literature search strategy.

Multimedia Appendix 2 Summary tables.

Multimedia Appendix 3 Forest plots of studies reported on intervention effect on patient outcomes.

Multimedia Appendix 4 Funnel plots of studies reported on intervention effect on patient outcomes.

Multimedia Appendix 5 Forest plots of studies reported on intervention effect on family member outcomes.

Multimedia Appendix 6 Funnel plots of studies reported on intervention effect on family member outcomes.

Multimedia Appendix 7 PRISMA checklist.

## Abbreviations

CONSORT: Consolidated Standards of Reporting Trials
HADS: Hospital Anxiety and Depression Scale
JBI: Joanna Briggs Institute
PHQ-9: Patient Health Questionnaire-9
PRISMA: Preferred Reporting Items for Systematic Reviews and Meta-Analyses
RCT: randomized controlled trial
STAI: State-Trait Anxiety Inventory
TREND: Transparent Reporting of Evaluations with Nonrandomized Designs
