# Supplementary material for: Digital Health Psychosocial Intervention in Adult Patients With Cancer and Their Families: Systematic Review and Meta-Analysis
Source: JMIR Cancer. 2024 Feb 5;10:e46116. doi: 10.2196/46116 (PMC10877499; doi:10.2196/46116)
Supplement: Multimedia Appendix 1 [file cancer_v10i1e46116_app1.docx]

Supplement: Search Strategies

**PubMed:** ("Social Media"[Mesh] OR "social media" OR "internet"[Mesh] OR "internet" OR "mobile applications"[Mesh] OR "mobile applications" OR "text messaging"[mesh] OR "text messaging" OR "cell phone use"[Mesh] OR "cell phone use” OR "cell phone"[Mesh] OR "cell phone"OR "virtual reality"[MeSH] OR "virtual reality" OR "web application" OR "web-based" OR "mobile application" OR "app-based" OR "internet application" OR "social media" OR "telemedicine"[MeSH] OR "telemedicine" OR "mhealth" OR "telemedicine"[MeSH] OR "telemedicine" OR "ehealth" OR "smartphone"[MeSH] OR "smartphone" OR "telemedicine"[All Fields] OR "ipad-based" OR "YouTube" OR "vlog" OR "FACEBOOK" OR "TWITTER" OR "INSTAGRAM" OR "text messaging") AND ("Neoplasms"[Mesh] OR cancer[sb]) NOT ( ("prevention" OR "screening") ) AND (English[lang] AND "adult"[MeSH])

**Cochrane Library:**

#1 MeSH descriptor: [Social Media] explode all trees

#2 MeSH descriptor: [Cell Phone] explode all trees

#3 MeSH descriptor: [Internet] explode all trees

#4 MeSH descriptor: [Mobile Applications] explode all trees

#5 MeSH descriptor: [Telemedicine] explode all trees

#6 MeSH descriptor: [Virtual Reality] explode all trees

#7 MeSH descriptor: [Smartphone] explode all trees

#8 MeSH descriptor: [Neoplasms] explode all trees

#9 Cancer

#10 Prevention

#11 MeSH descriptor: [Mass Screening] explode all trees

#12 (#1 OR #2 OR #3 OR #4 OR #5 OR #6 OR #7)AND #8 NOT (#10 OR #11)

**Web of Science:** TI=("social media" OR "internet" OR "mobile app*" OR "text messaging" OR "cell phone" OR "cell phone use" OR "virtual reality" OR "web app*" OR "telemedicine" OR "mhealth" OR "ehealth" OR " smartphone" OR "iPad" OR "facebook" OR "twitter" OR "instagram") AND TI=("cancer" OR "neoplasms" OR "malignancy") NOT TS= ("prevention" OR "screening") AND TS= clinical trial* OR TS=research design OR TS=comparative stud* OR TS=evaluation stud* OR TS=controlled trial* OR TS=follow-up stud* OR TS=prospective stud* OR TS=random* OR TS=placebo* OR TS=(single blind*) OR TS=(double blind*)

**CINAHL:** ( AB ( ((MH "Virtual Reality+") OR (MH "Cellular Phone+") OR (MH "Text Messaging+") OR (MH "Mobile Applications") OR (MH "Internet+") OR (MH "Social Media+")OR "youtube" OR "instagram" OR (MH "Twitter") OR "twitter" OR (MH "Facebook") OR "ipad" OR "ehealth" OR "mhealth" OR (MH "Telemedicine+") ) AND AB ( (MH "Neoplasms+") OR "neoplasms" OR “cancer” OR “malignancy”) ) ) NOT ( ("prevention" OR "screening") )

**EMBASE:** ('malignant neoplasm'/exp/mj OR 'malignant neoplasm'/mj OR 'neoplasm'/exp/mj OR 'neoplasm'/mj OR 'cancer'/exp/mj OR 'cancer'/mj) AND (('web application' OR 'web based intervention'/exp/mj OR 'web based intervention'/mj OR 'web-based' OR 'mobile application'/exp/mj OR 'mobile application'/mj OR 'internet'/exp/mj OR 'internet'/mj) AND application OR 'social media'/exp/mj OR 'social media'/mj OR 'virtual reality'/exp/mj OR 'virtual reality'/mj OR 'mobile phone'/exp/mj OR 'mobile phone'/mj OR 'smartphone'/exp/mj OR 'smartphone'/mj OR 'telemedicine'/exp/mj OR 'telemedicine'/mj OR 'mhealth'/exp/mj OR 'mhealth'/mj OR 'ehealth'/exp/mj OR 'ehealth'/mj OR 'tablet computer'/exp/mj OR 'tablet computer'/mj OR 'ipad'/exp/mj OR 'ipad'/mj OR 'youtube'/exp/mj OR 'youtube'/mj OR 'facebook'/exp/mj OR 'facebook'/mj OR 'twitter'/exp/mj OR 'twitter'/mj OR 'instagram' OR 'text messaging'/exp/mj OR 'text messaging'/mj) NOT ('prevention' OR 'screening') AND ([article]/lim OR [article in press]/lim) AND [english]/lim AND ([embase]/lim OR [embase classic]/lim)

**ProQuest Dissertations & Theses Global:** ab("web application" OR "web-based" OR "web based" OR "mobile application" OR "app-based" OR "internet application" OR "social media" OR "Virtual reality" OR "mHealth" OR "eHealth" OR "Mobile technology" OR "cell phone" OR "smartphone " OR "telemedicine" OR "ipad based" OR "iPad" OR "computer-based" OR "YouTube" OR "vlog" OR "FACEBOOK" OR "TWITTER" OR "INSTAGRAM" OR "text messaging") AND ab(cancer OR neoplasms OR malignancy) NOT(prevention OR screening)

**PsycINFO**: ab("web application" OR "web-based" OR "web based" OR "mobile application" OR "app-based" OR "internet application" OR "social media" OR "Virtual reality" OR "mHealth" OR "eHealth" OR "Mobile technology" OR "cell phone" OR "smartphone " OR "telemedicine" OR "ipad based" OR "iPad" OR "computer-based" OR "YouTube" OR "vlog" OR "FACEBOOK" OR "TWITTER" OR "INSTAGRAM" OR "text messaging") AND ab(cancer OR neoplasms OR malignancy) NOT(prevention OR screening)
