# Supplementary material for: Digital Health Psychosocial Intervention in Adult Patients With Cancer and Their Families: Systematic Review and Meta-Analysis
Source: JMIR Cancer. 2024 Feb 5;10:e46116. doi: 10.2196/46116 (PMC10877499; doi:10.2196/46116)
Supplement: Multimedia Appendix 2 [file cancer_v10i1e46116_app2.docx]

**Table S1.** Summary of selected study characteristics.

| First author (year) | Country | Randomi-zation method | Recruitment / attrition rate (%) | Sample size | Sample characteristics  (age, diagnosis, relationship to patient) | Appraisal composite/applicable score |
| --- | --- | --- | --- | --- | --- | --- |
| Randomized controlled trials (n=48) | | | | | | |
| Admiraal (2017) [35] | Netherlands | Block | 67.1/13.7 | IG: 70; CG:69 | Patients’ age (m, sd): IG: 53.1 (9.8) CG: 53.2 (8.5); breast cancer | 7/0.64 |
| Applebaum (2017) [36] | US | Simple | nr/nr | IG: 42; CG:42 | Caregivers’ age (m, sd): IG: 48.3 (11.4); CG: 51.9 (11.2). Mixed cancer diagnosis; relationship to patient: IG: parent 7, spouse 25, child 6, sibling 1, other 2; CG: parent 4, spouse 26, child 7, sibling 2, other 3 | 6/0.55 |
| Baker (2011) [37] | US | Block | nr/8 | IG1 (CHESS Information): 118; IG2 (CHESS information & support): 109; IG3 (full CHESS): 111; CG: 112 | Patients’ age (m, sd): IG1: 52.2 (9.8); IG2: 50.6 (10.8); IG3: 50.9 (9.0), CG: 52.3 (10.2). breast cancer | 8/0.67 |
| Berry (2013) [39] | US | Simple | nr/5.5 | IG: 266; CG: 228 | Patients’ age (range): IG: 45-86; CG: 40-84. Prostate cancer | 10/0.83 |
| Berry (2014)  [40] | US | Block | nr/17 | IG: 374; CG:378 | Patients’ age (range): IG: 22-86; CG: 19-88. Mixed cancer diagnosis | 8/0.73 |
| Boele (2018) [41] | Netherlands | Simple | nr/nr | IG: 45; CG1 (glioma waiting list): 44; CG2 (normal control): 26 | Patient’s age (m,sd): IG: 43.58 (11.69); CG1: 46.43 (12.28); CG2: 52.81 (9.28). Glioma | 6/0.50 |
| Børøsund (2014) [42] | Norway | Stratified | nr/14 | IG1 (WebChoice) 64; IG2 (IPPC): 45; CG: 69 | Patients’ age (range): IG1: 37-79; IG2: 31-66; CG: 36-69. Breast cancer | 8/0.73 |
| Braamse (2016) [43] | Netherlands | Stratified | 61.1/37 | IG: 47 CG: 48 | Patient’s age (m,sd): IG: 53.3 (8.7), CG: 55.5 (8.7). Hematological malignancies | 8/0.75 |
| Carpenter (2014) [44] | US | Simple | 93.1/12 | IG: 71; CG: 61 | Patient’s age (m, SD): total 50.9 (9.9). Breast cancer | 8/0.62 |
| Chambers (2018) [45] | Australia | Block | 12.7/  IG:47.6 CG: 44.3 | IG: 79; CG: 84 | Patients’ age (m): total sample 57. Colorectal, breast and melanoma | 7/0.64 |
| Classen (2013) [46] | Canada | NR | nr/14.8 | IG: 13; CG: 14 | Patient’s age (m, range): IG: 39.9 (29-58), CG: 44.6 (28-59). Gynecological cancers | 7/0.54 |
| Compen (2018) [47] | Netherlands | Stratified | IG1: 31.4 / 16.9; IG2: 36.7/ 16.7 CG: 31.8 / 10.3 | IG1 (MBCT): 77; IG2(eMBCT): 90; CG: 78 | Patient’s age (m, SD): IG1: 52.1(11.4); IG2: 52.4(10.7), CG: 50.4(9.9). Mixed cancer diagnosis | 11/0.85 |
| Diefenbach (2012) [48] | US | NR | 75/21 | IG1 (PIES tailored): 39; IG2 (PIES not tailored): 28; CG:24 | Patient’s age (m, SD): IG1: 60.03 (7.77); IG2: 62.81 (8.01); CG: 64.16 (8.35). Prostate cancer | 6/0.50 |
| DuBenske (2014) [49] | US | Stratified | nr/nr | IG: 144; CG:141 | Caregiver’s age (m, sd): IG: 56.56 (12.86); CG: 54.57 (12.21). Lung cancer. Spouse/partner in IG: 91; CG: 86 | 8/0.73 |
| Duffecy (2013) [50] | US | NR | 34/22.6 | IG1 (III+ Internet Support Group): 15; IG2 (III): 16 | Patient’s age (m): total: 50. Mixed cancer diagnosis | 5/0.45 |
| Foley et al. (2016) [52] | Ireland | NR | 83 / 0 | IG: 13; CG:26 | Patient’s age (median): IG: 54, CG:52. Breast cancer | 5/0.42 |
| Foster (2016) [53] | UK | Block | 41 / 36 | IG: 85; CG:78 | Patient’s age (m, SD): IG: 58.1(10.7) CG: 57.5 (9.1). Mixed cancer diagnosis | 6/0.55 |
| Freeman (2015) [54] | US | Stratified | nr/13.6 | IG1 (Live delivery): 48; IG2 (Telehealth delivery): 23; CG: 47 | Patient’s age (m, SD): IG1 55.44 (8.08); IG2: 55.57 (9.88); CG: 55.28 (7.90). Breast cancer | 7/0.54 |
| Giesler (2017) [55] | Germany | NR | 94.2/nr | IG: 103; CG: 109 | Patient’s age (m, SD): IG:54.5 (11.8); CG: 53.6 (10.5). Colorectal cancer | 6/0.55 |
| Greer (2019) [57] | US | Stratified | nr/11.7 | IG: 72; CG: 73 | Patient’s age (m, SD): IG: 55.86 (10.08); CG: 57.03(12.42). Mixed cancer diagnosis | 8/0.67 |
| Green (2019) [56] | US | Block | nr/nr | IG: 87; CG: 89 | Patient’s age (m, SD): IG: 61.38 (7.79) CG: 64.56 (7.22). Prostate cancer | 5/0.38 |
| Gustafson (2013) [59] | US | Block | nr/35.1 | IG: 144; CG: 141 | Patient’s age (m, SD): IG: 62 (10.6); CG: 61.4 (9.7). Non-small cell lung cancer | 6/0.55 |
| Gustafson (2008) [60] | US | Stratified | 83/IG1: 10, IG2: 12 CG: 10 | IG1 (Internet): 91; IG2 (CHESS): 83; CG: 83 | Patients age not reported. Breast cancer | 7/0.64 |
| Hawkins (2011) [61] | US | Stratified | nr/5.3 | IG1 (full CHESS): 111; IG2(mentor only): 106; IG3 (Full CHESS+mentor): 105 CG: 112 | Patient’s age (m, sd): IG1: 50.9 (9.0), IG2: 53.9 (10.9); IG3: 52.7 (9.4); CG: 52.3 (10.2). Breast cancer | 8/0.67 |
| Høybye (2010) [62] | Denmark | Block | nr/IG: 22, CG: 15 | IG: 361; CG: 433 | Patient’s age (m): IG: 53, CG: 55. Mixed cancer diagnosis | 7/0.58 |
| Huberty (2019) [63] | US | Simple | 44.2/26.6 | IG1 (10% Happier app follow by Calm): 33; IG2 (Calm app follow by 10% Happier app): 32; IG3 (control follow by 10% Happier app): 35; IG4 (control follow by Calm app): 28 | Patients’ age (m,sd): total sample 58 (12). Myeloproliferative Neoplasm | 5/0.45 |
| Hummel (2017) [64] | Netherlands | Stratified | 4.4/ 10.7 | IG: 84; CG: 85 | Patient’s age (m, SD): IG 51.1 (7.2); CG: 50.5 (6.8). Breast cancer | 9/0.75 |
| Jibaja-Weiss (2011) [65] | US | Block | 86.8/IG: 13.7 CG: 20 | IG: 51; CG: 49 | Patient’s age (m, SD): IG: 49.5 (10.3); CG: 52.4 (12). Breast cancer | 7/0.58 |
| Manne (2016) [72] | US | Simple | 56.7/22 | IG: 31; CG: 24 | Patient’s age (m, SD): IG: 51.7 (11.1) CG: 48.2 (9.7). Breast cancer | 9/0.75 |
| McCabe (2013) [73] | Ireland | Block | nr/9.5 | IG: 96; CG:103 | Patients’ age not reported. Hematological malignancies or aplastic anemia | 6/0.50 |
| Mohammad (2019) [76] | Jordan | Simple | nr/5 | IG: 40; CG: 40 | Patient’s age (m, SD): total: 51.99 (10.34). Breast cancer | 9/0.75 |
| Owen (2017) [77] | US | Simple | 49.2/33.3 | IG: 176; CG: 171 | Patient’s age (m, SD): I IG: 52.9 (10.7); CG:53.3 (11.1). Mixed cancer diagnosis | 8/0.67 |
| Oyama (2000) [78] | Japan | Simple | nr/nr | IG: 15; CG: 15 | Patient’s age (m): IG: 55.7, CG: 51.2. Mixed cancer diagnosis | 6/0.50 |
| Pfeifer (2014) [79] | US | Simple | nr/7 | IG: 48; CG: 38 | Patient’s age (m, SD): IG: 60.73 (10.2); CG: 59.67 (11.8). Head and neck | 5/0.42 |
| Rosen (2018) [80] | US | Stratified | nr/34 | IG: 57; CG: 55 | Patient’s age (m, SD): IG: 51.40(10.73); CG: 53.22(9.91). Breast cancer | 8/0.73 |
| Ruland (2013) [81] | Norway | Stratified | 73/24.6 | IG: 162; CG: 163 | Patient’s age (m, SD): IG: 56.9 (10.7); CG: 56.4 (11.5). Breast and prostate cancer | 8/0.73 |
| Ryha¨nen (2013) [82] | Finland | Stratified | 32.7/8.16 | IG: 48; CG: 50 | Patient’s age (m): IG: 54.4; CG: 55.7. Breast cancer | 6/0.46 |
| Syrjala (2018) [90] | US | Stratified | 58/17 | IG1(Inspire + PST): 108; IG2 (PST): 114; CG: 115 | Patient’s age (m, SD): IG1: 51 (12); IG2: 50 (13); CG: 51 (11). Survivors after hematopoietic cell transplantation. | 6/0.55 |
| Schover (2012) [85] | US | Stratified | nr/IG1: 33; IG2:25; IG3: 39; CG: 9 | IG1 (face-to-face):40; IG2 (Web-based 1):41; IG3 (Web-based 2):43; CG:48 | Patient’s age (m, SD): IG1: 64 (8); IG2: 64 (7); IG3: 64 (8); CG: NR. Prostate cancer | 7/0.54 |
| Steel (2016) [89] | US | Block | 77/nr | IG: 144; CG: 117 | Patient’s age (m, SD): total: 61(11). Liver tumor | 8/0.62 |
| van den Berg(2015) [91] | Netherlands | Stratified | nr/17.3 | IG: 70; CG: 80 | Patient’s age (m, SD): IG: 51.44(8.30) CG: 50.18(9.15). Breast cancer | 10/0.91 |
| Villani (2018) [93] | Italy | NR | 72.5/nr | IG: 15; CG: 14 | Patient’s age (m, SD): total: 62.76 (6.19). Breast cancer | 7/0.54 |
| Washington (2018) [94] | US | NR | nr/nr | IG: 42; CG: 41 | Caregivers’ age (m, SD): total 51.5 (12.8). Patient’s age (m, SD): total: 60.4 (12.8). Mixed cancer diagnosis. Caregivers included Spouse or partner:44; Adult child:22; Sibling:7; Parent:3; Friend:3; Other:4 | 6/0.46 |
| White (2018) [95] | Australia | Simple | 76/24 | IG: 202; CG: 177 | Patient’s age (m, SD): IG: 43.6 (5.0); CG: 43.9 (5.3). Breast cancer | 7/0.64 |
| Willems (2017) [96] | Netherlands | Simple | nr/11.5 | IG: 252; CG: 240 | Patient’s age (m, SD): IG: 56.26 (10.84); CG:56.28 (11.45). Mixed cancer diagnosis | 9/0.75 |
| Wise (2018) [97] | US | Stratified | 33/22 | IG: 59; CG: 51 | Patient’s age (m, SD): IG: 59 (8.5); CG: 57(9.1). Mixed cancer diagnosis | 8/0.67 |
| Yanez (2015) [98] | US | Stratified | 31.3/  IG:14.3 CG:13.9 | IG: 37; CG: 37 | Patient’s age (m, SD): total: 68.84 (9.23). Prostate cancer | 6/0.50 |
| Yun (2012) [99] | Korea | Stratified | nr/nr | IG: 136; CG: 137 | Patient’s age (percentage of >45 y.o.): IG: 52.2; CG: 54.7. Mixed cancer diagnosis | 8/0.67 |
| Quasi-experimental studies (n=17) | | | | | | |
| Becker (2017) [37] | US | NA | nr/10 | IG: 20 | Patient’s age (m, SD): Total: 53.1 (8.2). Breast cancer. | 7/0.78 |
| Espinoza (2012) [51] | Spain | NA | nr/36.4 | IG: 33 | Patient’s age (m, SD): total: 62.1(10.77). Mixed cancer diagnosis | 6/0.67 |
| Gustafson (2017) [58] | US | NA | nr/33.6 in CHESS; 38.1 in CHESS + Clinician report | IG1: 117; IG2: 118 | Caregiver’s age (m, SD): IG1: 55.73 (13.02); IG2: 56.36 (13.39). Patients with Breast cancer. N=150 (69.3%) were spouses or partners. | 7/0.78 |
| Jimenez (2018) [66] | Australia | NA | nr/nr | IG: 19; CG: 18 | Patient’s age (m, SD): NR. Breast cancer | 7/0.78 |
| Kazer (2011) [67] | US | NA | nr/33 | IG: 9 | Patient’s age (m): total: 72. Prostate cancer | 7/0.78 |
| Kinner (2018) [68] | US | NA | 32/39 | IG: 28 | Patient’s age (m, SD): total 58.89(6.87). Ovarian cancer | 5/0.71 |
| Kubo (2018) [69] | US | NA | 17-87/32.1 in patient; 35.7 in caregiver | IG: 28 patients and 14 caregivers | Patient’s age (m, range): total 64.7 (48-78). Caregiver’s age (m, range): total 58.8 (38-73). Mixed cancer diagnosis. Spouse 6; child 2 friend 1 | 7/0.78 |
| Kuijpers (2016) [70] | Netherlands | NA | 35.4/13 | IG: 92 | Patient’s age (m, SD): total: 49.5(11.4). Breast cancer | 5/0.63 |
| Lengacher (2018) [71] | US | NA | nr/13 | IG: 15 | Patient’s age (m, SD): total: 57 (9). Breast cancer | 6/0.67 |
| McCarthy (2018) [74] | US | NA | nr/nr | IG: 18 | Patient’s age (m, SD): total: 57.72 (6.49). Breast cancer | 7/0.78 |
| Mihuta (2018) [75] | Australia | NA | nr/IG1 (cancer group): 8; IG2 (non-cancer group): 24; CG: 12 | IG1: 16; IG2: 23 CG: 20 | Patient’s age (m, SD): IG: 45.4 (10.3); CG1: 47.6 (10.0); CG2: 45.9 (7.4). Mixed cancer diagnosis | 6/0.67 |
| Schneider (2007) [83] | US | Simple | 64/18.7 | IG: 107; CG: 107 | Patient’s age (m, SD): Total: 53.97 (10.89). Mixed cancer diagnosis | 8/0.78 |
| Schneider (2004) [84] | US | Simple | 88/0 | IG: 20 | Patient’s age (m, SD): 42.6 (7.9). Breast cancer | 7/0.89 |
| Shepherd (2006) [86] | Australia | NA | 61/26 | IG: 25 | Patient’s age (m, SD): total: 53 (10.53), diagnosis not reported | 6/0.67 |
| Smith (2018) [87] | US | NA | nr/nr | IG: 31 | Patient’s age (m, SD): total: 55.9 (9.2). Diagnosis not reported | 8/0.89 |
| Song (2015) [88] | US | NA | 51/15.4 | IG: 26 patients and 26 partners | Patient’s age (m, SD): total: 62.95 (8.22). Partner’s age (m, SD): 59.32 (10.67). Prostate cancer | 7/0.78 |
| VanDen Brink (2007) [92] | Netherlands | NA | IG: 66 / 10.3; CG: 85 /11.7 | IG: 39; CG: 145 | Patient’s age (m): IG: 59 CG: 61.Head and neck cancer | 6/0.67 |

Note. NR=not reported; NA=not applicable; IG=intervention group; CG=control group; CHESS=Comprehensive Health Enhancement Support System; PST=Problem-solving treatment; Inspire=Internet-based survivorship program with information and resources; III= Individual Internet Intervention; IPPC=Internet-based patient provider communication service; WebChoice=a Web-based illness management system for breast cancer patients (IPPC included); MBCT=Mindfulness-based cognitive therapy; eMBCT= Individual Internet-based MBCT; PIES=Prostate Interactive Educational System; Possible appraisal composite scores ranged from 0-9 for quasi-experimental studies and 0-13 for RCTs, with higher scores indicating less risk of bias and better study quality. The applicable score (range 0-1) was calculated by dividing the composite score by the maximum score possible after subtracting any “not applicable” responses.

**Table S2.** Summary of intervention characteristics.

| First author (year) | Theoretical or conceptual framework | Partici-pants | Type of intervention | N of arms | Control | Intervention component | Delivery mode | Prescribed dosage (number of sessions, frequency, access period) | Received dosage | Interven-tionist | Follow-ups |
| --- | --- | --- | --- | --- | --- | --- | --- | --- | --- | --- | --- |
| Admiraal (2017) [35] | Problem-solving orientation | Patient | Tailored | 2 | Usual care | Problem-solving therapy;  Tailored psychoeducation | Web-based | Visit the program at least once during the first 7 days; access the program for 12 weeks | Number of logins: range = 0-7, median = 2; 61% of the patients log in more than once | Self-delivered | 6, 12 weeks  Post-baseline |
| Applebaum (2017) [36] | Meaning‐Centered Psychotherapy | Caregiver | Standardized | 2 | Usual care | Meaning‐Centered Psychotherapy | Web-based | 5 webcasts, access the website within 14 weeks post-baseline | NR | Self-delivered | End of intervention,  2-3 months |
| Baker (2011) [37] | None | Patient | Standardized & tailored | 4 | Active | IG1: CHESS information.  IG2: CHESS information & support.  IG3: full CHESS. | Web-based | NR | NR | Self-delivered | 2, 6, 12, 24 weeks post-baseline |
| Becker* (2017) [38] | Pender’s Model of Health Promotion, Bandura’s Social Cognitive Theory | Patient | Standardized | 1 | NA | Cognitive exercise training program | Com-puter based | 6 weekly sessions, 90 minutes each; practice 3-4 times (45 minutes each time) per week | 65% attended at least 5 of 6 sessions; practice M = 33 minutes per week (range = 2-523 minutes) | Master’s level nurse | 1-2 weeks after intervention |
| Berry (2013) [39] | Ottawa Decision Support Framework | Patient | Standardized& tailored | 2 | Usual care | Customized education | Internet-based | NR | NR | Self-delivered | 1-month,  6-month post-baseline |
| Berry (2014) [40] | Quality Health Outcomes Model | Patient | Tailored | 2 | Active | Patient education and coaching | Internet-based | NR | A median access rate of 4 (range = 2-4) at study time points and of 1 (range = 0-8) at voluntary times | Self-delivered | 3-6 weeks after starting treatment,  2 weeks later, 2-4 weeks after treatment |
| Boele (2018) [41] | None | Patient | Standardized | 3 | Usual care | Problem-Solving Therapy, disease specific information and examples | Internet-based | 5 weekly modules | 85% completed introduction; 77%, 52%, 40%, 37% and 35% for modules 1-5 respectively. | Psychologist, nurse, or a trained psychology student | End of intervention, 12 weeks post-intervention |
| Børøsund (2014) [42] | None | Patient | Standardized& Tailored | 3 | Usual care | IG1(WebChoice);  seek help from health care provider  IG2(IPPC): Self- management support, information and resources,  online forum,  electronic diary | Internet-based | None, entirely voluntary | IG1: Median of total visits = 9.5 (range = 1-17), 61% sent e-messages, 20% posted in the discussion forum, 37% posted their own blogs or reading other blogs; advice were accessed (range = 0-62, median = 5)  IG2: Median of total visits = 7 (range = 2-41), 39% sent e-messages | Self-delivered | 2-, 4-, and 6-month post-baseline |
| Braamse (2016) [43] | Principles of Problem-Solving Treatment | Patient | Standardized& Tailored | 2 | Usual care | Information, individualized support | Internet-based | Step 1: watchful waiting, no dose. Step 2: 10 hours of module learning & 5 weekly brief emails. Step 3: no dose | 41.7% completed at least 4 of 5 modules | Step 2:  Psychologist | 13-, 30-, 42-weeks post-baseline |
| Carpenter (2014) [44] | Cognitive Behavioral Stress Management | Patient | Standardized | 2 | Usual care | Cognitive-behavioral strategies | Web-based | 10 chapters, weekly | 29% completed all 10 chapters. Visits to pages: M = 159.8 (SD = 93.1); Days signed on: M = 13.3 (SD = 8.8) | Self-delivered | End of intervention, 20 weeks post-baseline |
| Chambers (2018) [45] | None | Patient | Tailored | 2 | Active | Cognitive-behavioral therapy | Web-based | 6 weekly sessions, access for 12 months | 10% accessed all 6 cores, with 47% not accessing any cores; 28% accessed ≥3 cores | Self-delivered | 2-month post-baseline |
| Classen (2013) [46] | None | Patient | Standardized& Tailored | 2 | Usual care | Peer-support, psychoeducation | Web-based | Post on discussion forum at least once a week | 23% post ≥12 times, 31% in Group 1 and 36% in Group 2 joined in real time chat | Psychologists | End of intervention, 8-month post-baseline |
| Compen (2018) [47] | None | Patient | Standardized& Tailored | 3 | Usual care | Mindfulness-Based Cognitive Therapy (MBCT) | Internet-based | 8 weekly 2.5 hour group sessions, 6 hr silent day, & daily home practice for 8 weeks | MBCT group: 92.2% completed ≥4 sessions (M = 7.9; SD = 1.3); daily practice M = 30.6; SD = 26 minutes  eMBCT group: 78.9% completed ≥4 sessions (M = 8.6; SD = 1.2); daily practice of M = 28.7; SD = 29.3 minutes | Mindfulness-based therapists | Immediately after completion of the intervention |
| Diefenbach (2012) [48] | Self-Regulation Theory | Patient | Tailored | 3 | Active | Information, support group | Internet-based | 45 minutes | NR | Self-delivered | End of intervention |
| DuBenske (2014) [49] | Model of Coping Self-efficacy | Caregiver | Standardized& Tailored | 2 | Active | Information, communication, assessment of emotional and health status, and links and material addressing specific concerns | Web-based | Voluntary rather than assigned | Logins: M = 14.6 (SD = 25.1), pages: M = 293 (SD = 482.1), length of time: 177.7 minutes (SD = 24.5) | Self-delivered | 2-, 4-, 6-, 8 months post-baseline |
| Duffecy (2013) [50] | Cognitive behavioral principles | Patient | Standardized | 2 | Active | Cognitive-behavioral treatment for depression,  Self-monitoring and discussion board | Web-based | III: 16 sessions, 10-15 minutes each; 2 times a week; access for 8 weeks  ISG: access for 8 week | III+ISG group: login M = 20.8, SD = 7.7;  III group: login M = 12.5; SD = 12.5 | Self-delivered | Mid-intervention; end of intervention |
| Espinoza (2012) * [51] | None | Patient | Standardized | 1 | NA | Virtual reality | Virtual reality | 4 sessions, 30 minutes each | 120 minutes | Self-delivered | End of intervention |
| Foley (2016) [52] | None | Patient | Tailored | 2 | Usual care | Tailored information | App | NR | NR | Self-delivered | 1 day, 7 day post-intervention |
| Foster (2016) [53] | Self-efficacy Theory | Patient | Standardized & Tailored | 2 | Active | Psychoeducation, patients’ stories, self-monitoring, feedback, web links | Web-based | 5 weekly sessions, including 2 mandatory and 3 optional sessions. | 71% attend ≥2 sessions, 60% logged on to 4 sessions, 43% completed 5 sessions | Self-delivered | 6-, 12-week (end of intervention) |
| Freeman (2015) [54] | None | Patient | Standardized | 3 | Usual care | Psychoeducation, mental imagery, feedback | Telehealth | 5 weekly 4-hour group sessions, 5 weekly 10-minute phone calls, 5 20-30 minutes guided imagery | Live delivery group: 85.4% attended all sessions.  Telemedicine delivery group: 94.7% attended all sessions | A licensed professional counselor, and a family medicine physician | 1-, 3-month post-intervention |
| Giesler (2017) [55] | None | Patient | Standardized & Tailored | 2 | Usual care | Experiential information | Web-based | NR | Website visit: M = 42.21, SD = 45.64; sessions accessed: M = 3.43, SD = 2.94; number of clicks: M = 40.15, SD = 42.14 | Self-delivered | End of intervention, 6 weeks post- baseline |
| Green (2019) [56] | None | Patient and caregiver | Standardized | 2 | Active | Information, Resources | App | Access the application for 6 weeks | NR | Community navigator + Self-delivered | End of intervention; 1-, 6-; 12-months post-baseline |
| Greer (2019) [57] | None | Patient | Standardized | 2 | Active | Tailored Cognitive Behavioral Therapy | App | 6 sessions, 20-30 minutes each; homework, 10-15 minutes each | 70.8% used app at least through session 5 | Self-delivered | 12 weeks post-baseline |
| Gustafson (2008) [60] | Self-Determination Theory | Patient | Standardized & Tailored | 3 | Active | Information, communication, decision services | Internet-based | The training averaging 58 minutes; access for 5 months | I: M = 58 minutes;  C: M = 51 minutes | Self-delivered | 2, 4, and 9 months post--baseline |
| Gustafson (2017) * [58] | None | Caregiver | Standardized | 2 | Active | Information, summary about symptom reported by caregiver | Interactive portal, eHealth | NR | NR | Self-delivered | 2-, 4-, 6-, 8-, 10-, 12-months post-baseline |
| Gustafson (2013) [59] | None | Patient and caregiver | Standardized & Tailored | 3 | Active | Information, communication, individual assessment of emotional and health status, links and material addressing specific health concerns | Web-based | Access the system for 25 months or 13 months after patient death | Patient: 50% used ≥once; 34.7% used 5 times; length of use: median = 146 minutes; number of pages viewed: median = 243; number of logins: median = 12  Caregiver: 73.4% used ≥once; 51.6% used 5 times; length of use: median = 103 minutes; number of pages viewed: median = 147; number of logins: median = 8 | Self-delivered | 2-, 4-, 6-, 8-month post-baseline |
| Hawkins (2011) [61] | None | Patient | Standardized & Tailored | 4 | Active | IG1: Information;  IG2: Information & support;  IG3: Information &  Support &coaching | Internet-based | CHESS: access for 6 months  Other groups: 10 calls, average of 15 minutes each | The average length of calls: CHESS + Mentor group 29 minutes, mentor-only group 23.8 minutes; for CHESS use, total time in the CHESS + Mentor condition was 553 minutes, CHESS-Only condition 619 minutes. | Information specialist | 6 weeks,  3-, 6-month  post-baseline |
| Høybye (2010) [62] | None | Patient | Tailored | 2 | Usual care | Peer-support group, rehabilitation program | Internet-based | 2-hour introductory lecture; access the Internet-based support group for 12 months | 60% of patient posted ≥2 messages online. The total number of messages posted ranged 2-241 in the Internet groups. | Self-delivered | 1-, 6- and 12-month post-baseline |
| Huberty (2019) [63] | None | Patient | Standardized | 4 | Active | IG1: The 10% Happier app: daily meditation.  IG2: Calm App: daily meditation + brief experiential practices. | App | 10 minutes meditation daily for 8 weeks | 10% Happier app: M = 31 (SD = 33) minutes per week.  Calm app: M = 71 (SD = 74) minutes per week. | Self-delivered | 5-week post-baseline, end of intervention |
| Hummel (2017) [64] | Cognitive Behavioral Therapy | Patient | Standardized & Tailored | 2 | Usual care | Cognitive behavioral therapy | Internet-based | 20 weekly sessions completed within 24 weeks, 90-120 minutes each. A minimum of 5 sessions is required. | 61.9% of women completed intervention (M = 22.1 weeks; SD = 4.5); 31.0% ended the CBT prematurely (M = 9.6 weeks; SD = 5.8), and 7.1% never started | Psychologist/sexologist | Intervention: 10 weeks post-baseline, end of intervention.  Control: 13-, 23-week post-baseline. |
| Jibaja-Weiss (2011) [65] | Ottawa Decision Support Framework | Patient | Standardized | 2 | Usual care | Interactive patient decision aid | Computer based | One session | NR | Case manager | 12 months post-baseline |
| Jimenez (2018) * [66] | none | Patient | Standardized | 2 | Usual care | Virtual environment | Virtual reality | 1 hour session | 1 hour session | Radiation therapist with VERT education experience | First week of treatment, the last week of treatment |
| Kazer (2011) * [67] | Mishel's Uncertainty in Illness Theory | Patient | Standardized | 1 | NA | Cognitive reframing and self-management strategies | Internet-based | Access the website for 5 weeks | Web page views 2–40 times, with an average of 20 page views per participant | Self-delivered | End of intervention; 5 weeks post-intervention |
| Kinner (2018) * [68] | Cognitive Behavioral Stress Management | Patient | Standardized & Tailored | 1 | NA | Mindfulness-based Stress Reduction | Web-based | 10 weeks group sessions, 1.5 to 2 hours each, at home practice | Attend group session: M = 8.79 (SD = 1.08). Home practice per week: M = 2.78; journal use per week: M = 2.34 | Clinical psychologist | End of intervention completion |
| Kubo (2018) * [69] | None | Patient and caregiver | Standardized | 1 | NA | Mindfulness: HEADSPACE | App | 30 daily 10-20 minutes sessions, optional 10-, 30-day courses. | NR | Self-delivered | End of intervention |
| Kuijpers (2016) * [70] | Social Cognitive Theory and theory of planned behavior | Patient | Standardized & Tailored | 1 | NA | Personalized information, patient reported outcome results to primary doctor, physical activity advice | Internet-based | Access for 4 months | Range of number of logins = 0-62; duration = 2-38 minutes | Self-delivered | End of intervention |
| Lengacher (2018) * [71] | None | Patient | Standardized | 1 | NA | Mindfulness‐based Stress Reduction | App | 6 weekly sessions, 2 hrs each, practice for 15 to 45 minutes per day for 6 weeks. | The average practice time was 36 minutes per day | Self-delivered | End of intervention |
| Manne (2016) [72] | Decision Support Framework | Patient | Standardized | 2 | Active | Decision aid | Web-based | NR | 77.4% used decision aid | Self-delivered | End of intervention |
| McCabe (2013) [73] | None | Patient | Standardized & Tailored | 2 | Usual care | Virtual reality | Virtual reality | NR | 70% patients watched OW for ≥3 days per week, and 61% viewed OW for periods of up to 1 hour per day. | Self-delivered | Day of admission;  the day before  transplant; 7 days after transplant;  prior to discharge; 60-, 100- days, and 6-month post- transplant |
| McCarthy (2018) * [74] | Spielman 3P Model of Insomnia | Patient | Standardized & Tailored | 1 | NA | Cognitive-behavioral Therapy for Insomnia | Telehealth | 6 weekly sessions, 30-60 minutes each | 6 sessions, 30-60 minutes each | Advanced practice nurse | End of intervention |
| Mihuta (2018) * [75] | None | Patient | Standardized & Tailored | 3 | Usual care | Cognitive-behavioral Therapy | Web-based | 4 weekly session, 2 hours each | NR | Self-delivered | End of intervention, 3-month post-intervention |
| Mohammad (2018) [76] | None | Patient | Standardized | 2 | Usual care | Virtual reality | Virtual reality | 1 session | NR | Self-delivered | Before morphine, 15 minutes from the peak time effect |
| Owen (2017) [77] | Model of Stress and Appraisal Coping | Patient | Standardized & Tailored | 2 | Usual care | Social networking | Web-based | 12 weekly sessions; 90 minutes each | Total engagement 438 minutes per participant | Doctoral-level clinical psychology students | End of intervention |
| Oyama (2000) [78] | None | Patient | Standardized & Tailored | 2 | Usual care | VR, Aromatherapy | Virtual reality | 1 session, 20 minutes | NR | Self-delivered | End of intervention |
| Pfeifer (2014) [79] | None | Patient | Standardized & Tailored | 2 | Usual care | Self-management of symptom strategies | Telehealth | Response to algorithm questions daily for 6 weeks, 5-10 minutes each. | NR | Study staff | Midpoint of treatment; 3 wk after treatment |
| Rosen (2018) [80] | None | Patient | Standardized | 2 | Usual care | Mindfulness training: HEADSPACE | App | Minimum of one 10‐minute introductory session, daily use for 8 weeks | Engagement (minutes per day): M = 13.4, range = 10-40. Individual mindfulness sessions range = 3-20 minutes. | Self-delivered | 5-, 9-, 12-week post-baseline |
| Ruland (2013) [81] | None | Patient | Standardized & Tailored | 2 | Active | Symptom monitoring, tailored symptom self-management, Information and resources, support group, personal diary | Internet-based | Access to the system for 1 year, voluntary | 77% logon at least once, 64% used Web Choice (M = 60, range = 2-892); message nurse (range = 1-49; M = 6.2); posted on forum (range = 1-58; M = 10.15); average of 15 minutes on answering questions | Self-delivered & nurses answer questions | 3-, 6-, 9-, and 12- months post-baseline |
| Ryha¨nen (2013) [82] | Theory of empowering knowledge | Patient | Standardized | 2 | Usual care | Education | Internet-based | 1 session, 30 minutes (range = 10-60 minutes) | NR | Researcher, self-delivered | Have surgery, 1 day after surgery, first oncologist visit, before and after chemotherapy, before and after radiation, 1year after diagnosis |
| Schneider (2004) * [84] | Lazarus and Folkman’s Stress and Coping Model | Patient | Standardized | 1 | Usual care | Virtual reality distraction intervention | Virtual reality | One session which lasts the duration of chemo infusion | M = 42 minutes | Self-delivered | End of, 48-hour after chemothera-py |
| Schneider (2007) * [83] | Lazarus and Folkman’s Stress and Coping Model | Patient | Standardized | 2 | Usual care | Virtual reality distraction intervention | Virtual reality | One session which lasts the duration of chemo infusion | 45–90 minutes | Self-delivered | End of, 48-hour after chemotherapy x 2 cycles |
| Schover (2012) [85] | None | Patient and partner | Standardized & Tailored | 3 | Usual care | Cognitive-behavioral treatment,  a decision aid for choosing an ED treatment | Internet-based | 3 sessions, total of 3.5 hour; 2 booster telephone calls, 15-30 minutes each; access for 12 weeks | NR | Therapists | After treatment, 3-, 6-, and 12-month follow-up |
| Shepherd (2006) * [86] | None | Patient | Tailored | 1 | NA | Cognitive-behavioral techniques | Videoconference | 6 sessions, depends on need, weekly or biweekly | NR | Clinical psychologist | Post-test, 1 month follow-up |
| Smith (2018) * [87] | None | Patient | Standardized | 1 | NA | Cancer distress coaching, information and resources,  self-assessment of PTSD checklist and provides feedback, mind-body exercises | App | 4 modules, access for 8 weeks | Used the app 2.8 times per week (SD = 3.2). | Self-delivered | 4 weeks, 8 weeks post-baseline |
| Song (2015) * [88] | None | Patient and partner | Standardized | 1 | NA | Information about couple communication, symptom management strategies | Web-based | 7 modules, including 2 mandatory, 5 optional modules | Logins: couple M = 3.64 (SD = 1.68); patients M = 2.73 (SD = 1.2); partners, M = 2.68 (SD = 1.39). Time spent (minutes): couple M = 56.96 (SD = 39.74); patients M = 41.99 (SD = 26.21); partners M = 43.99 (SD = 43.69) | Self-delivered | Pre-, post- intervention |
| Steel (2016) [89] | None | Patient | Standardized & Tailored | 2 | Active | Psychoeducation, CBT, pharmacological strategies to manage symptoms | Web-based | Access website for 6 months, telephone contact every 2 weeks, in-person contact every 2 months | Total of 1491 page views times, total duration of 1813.9 minutes | Master’s level or PhD therapists trained in CBT | End of intervention |
| Syrjala (2018) [90] | None | Patient | Tailored | 3 | Active | IG1 (INSPIRE): Information and resources.  IG2 (PST): problem-solving treatment. | Internet-based | INSPIRE: Access website for 6 months  PST call: first session an hour, 3-7 sessions, 30 minutes each, every two weeks | INSPIRE: Median number of page views was 9, range = 0–23 PST: participants received 4.5 calls (SD = 2.8). | Inspire: Self-delivered,  PST calls: trained psychologist | End of intervention |
| van den Berg (2015) [91] | Transactional model of stress, Model of psychological well-being | Patient | Standardized | 2 | Usual care | Cognitive-behavioral treatment | Web-based | 16 weekly modules | Logins: range 0-45, duration: range 0-2,324 minutes, intervention component opened range 0-104 | Self-delivered | End of intervention, 6-, 10-month post-baseline |
| VanDen Brink (2007) * [92] | None | Patient | Standardized & Tailored | 2 | Usual care | Communicate (send messages) with professionals, information, peer-support (via a forum), home monitoring | Electronic health support system | Access to the telemedicine system for 6 weeks | Number of sessions per patient: M = 27 (SD = 18, range = 4–69), 12 minutes each session on average. Patient sent on average 4.5 messages. | Self-delivered | End of intervention, 3 month post-baseline |
| Villani (2018) [93] | NR | Patient | Standardized | 2 | Usual care | Stress inoculation training | Internet-based | 10 sessions, 25 minutes each | NR | Self-delivered | End of intervention, 3 months after intervention |
| Washington (2018) [94] | Relational/  Problem‐solving Model of Stress | Caregiver | Standardized & Tailored | 2 | Usual care | Problem‐solving therapy | Web-based | 3 weekly sessions | NR | Research nurse | Day 15, 30, 60 post-baseline |
| White (2018) [95] | None | Patient | Standardized | 2 | Usual care | Information and resources; diary | Web-based | Access the website for 6 months | Number of days: median = 1, range = 1-15. The total time spent on the website: median = 19, range = 1–315 minutes. | Self-delivered | 3-, 6-month post-baseline |
| Willems (2017) [96] | Problem-solving Therapy and Cognitive Behavioral Therapy | Patient | Tailored | 2 | Usual care | Self-management Problem-solving Therapy | Web-based | 8 modules, users can choose which modules they want to visit and can skip assignments, access for 6 months | Module used: M = 2.2 (SD = 1.58). 89.4% used ≥1 module, the average time between first login and last use was 10.67 weeks (SD = 6.78) | Self-delivered | End of intervention, 12-month post-baseline |
| Wise (2018) [97] | None | Patient | Tailored | 2 | Active | Stories telling, information and resources, social network | Web-based | One 20–30 minute phone call, one-hour interview; access website for 4 months | Average of 1.06 hours per week (range = 0-2) used social network, 45% in living story and 54% own resources used the website with visits 3.27 (range = 1-10) and 4.56 (range = 1-23) respectively. | Interview with researcher | 2-, 4-month post-baseline |
| Yanez (2015) [98] | None | Patient | Standardized | 2 | Active | Cognitive-behavioral stress management | Web-based | 10 weekly sessions, 90 minutes each | M = 6.59 sessions completed | Master’s level therapists | 6 months post-baseline |
| Yun (2012) [99] | Transtheoretical Model of Health Behavior Change, Social Cognitive Theory | Patient | Tailored | 2 | Usual care | Self-assessment, education, messaging, caregiver support, health monitoring | Web-based | Access for 12 weeks, includes 7 education areas | 83.1% completed 12-week course | Self-delivered | End of intervention |

Note. NR=not reported; NA=not applicable; IG=intervention group; CG=control group; CHESS=Comprehensive Health Enhancement Support System; VERT=Virtual Environment for Radiotherapy Training; OW=Open window virtual reality; PST=Problem-solving treatment; INSPIRE=Internet-based survivorship program with information and resources; III= Individual Internet Intervention; IPPC=Internet-based patient provider communication service; WebChoice=a Web-based illness management system for breast cancer patients (IPPC included); MBCT=Mindfulness-based cognitive therapy; eMBCT= Individual Internet-based MBCT; PIES=Prostate Interactive Educational System. *quasi-experimental study.
