# Supplementary material for: Digital Health Psychosocial Intervention in Adult Patients With Cancer and Their Families: Systematic Review and Meta-Analysis
Source: JMIR Cancer. 2024 Feb 5;10:e46116. doi: 10.2196/46116 (PMC10877499; doi:10.2196/46116)

Forest plot for the studies reported on intervention effects in patients

**Forest plot of studies reported on intervention effects on quality of life in patients (Overall effect, outcome measures 1: FACTB; 2: FACTG; 3: QLQ30; 4: SF36).**


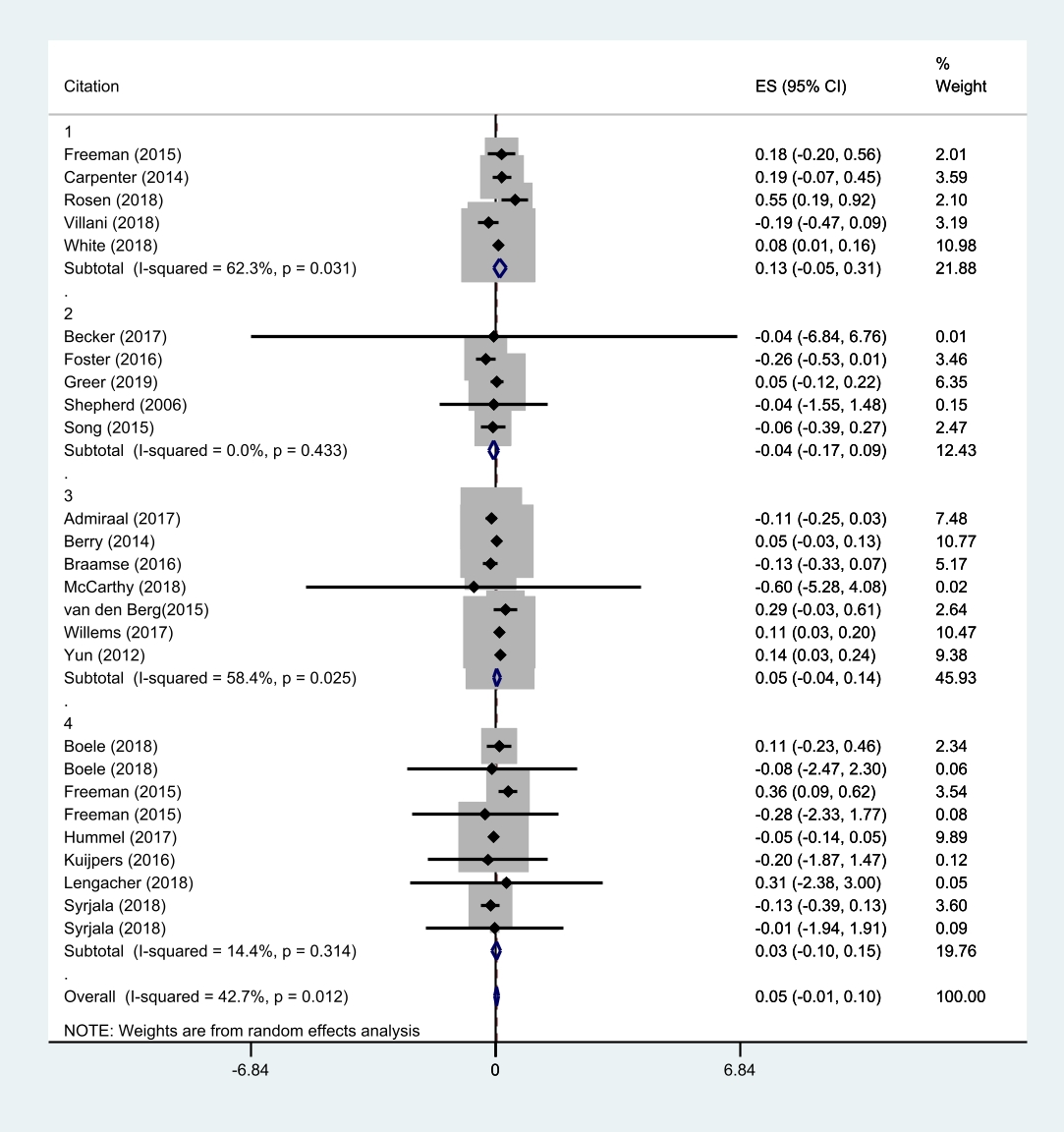


**Forest plot of studies reported on intervention effects on quality of life in patients (time-varying effect; 1=Immediate, 2=interim, 3=short-term, 4=medium-term).**


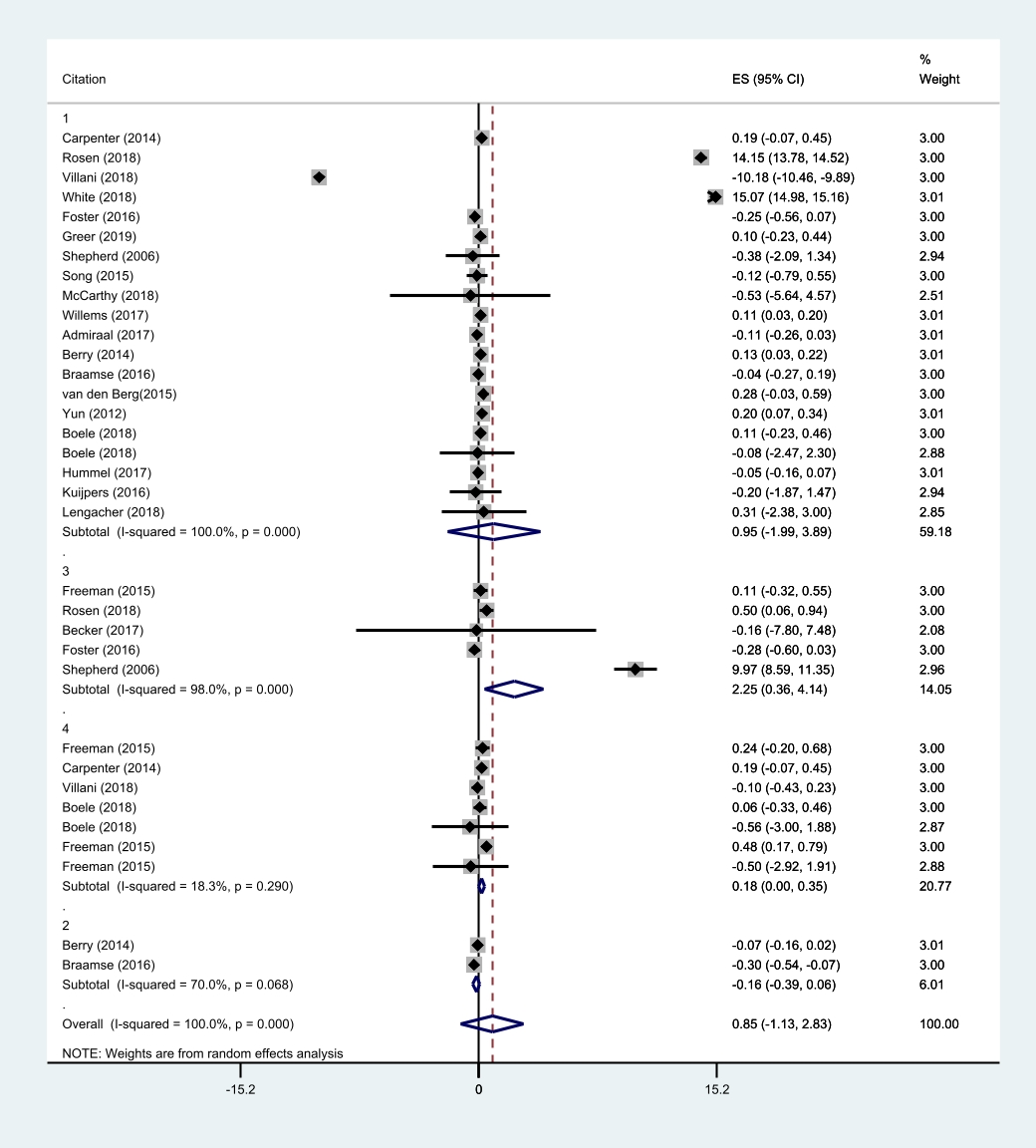


**Forest plot of studies reported on intervention effects on anxiety and depression measured by HADS total score (overall effect).**


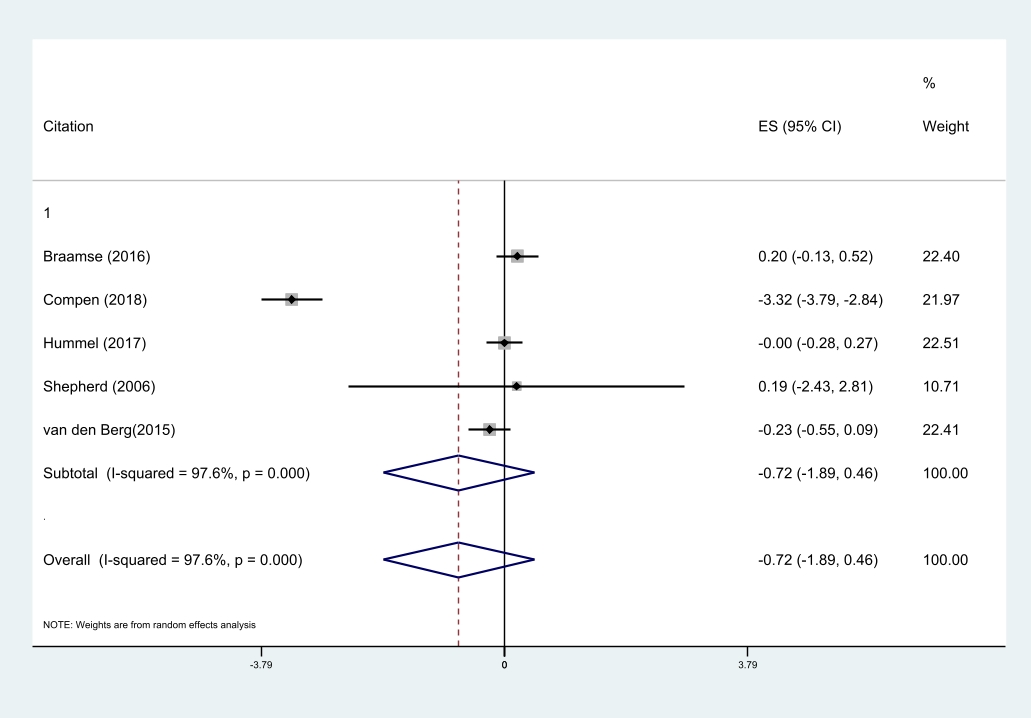


**Forest plot of studies reported on intervention effects on Anxiety and depression measured by HADS total score (time-varying effect; 1=Immediate, 3=short-term, 4=medium-term).**


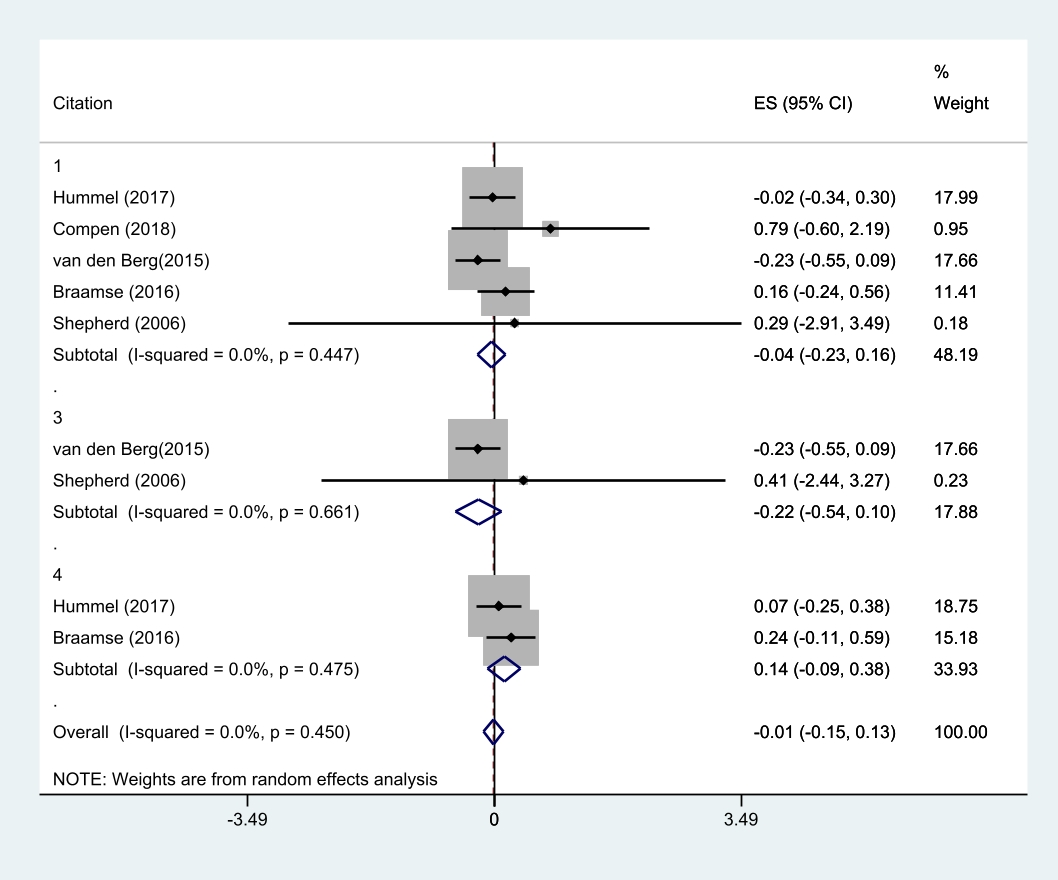


**Forest plot of studies reported on intervention effects on depression (overall effect, outcome measures: 1: HADS; 2: CESD; 3: PHQ9; 7: mixed).**


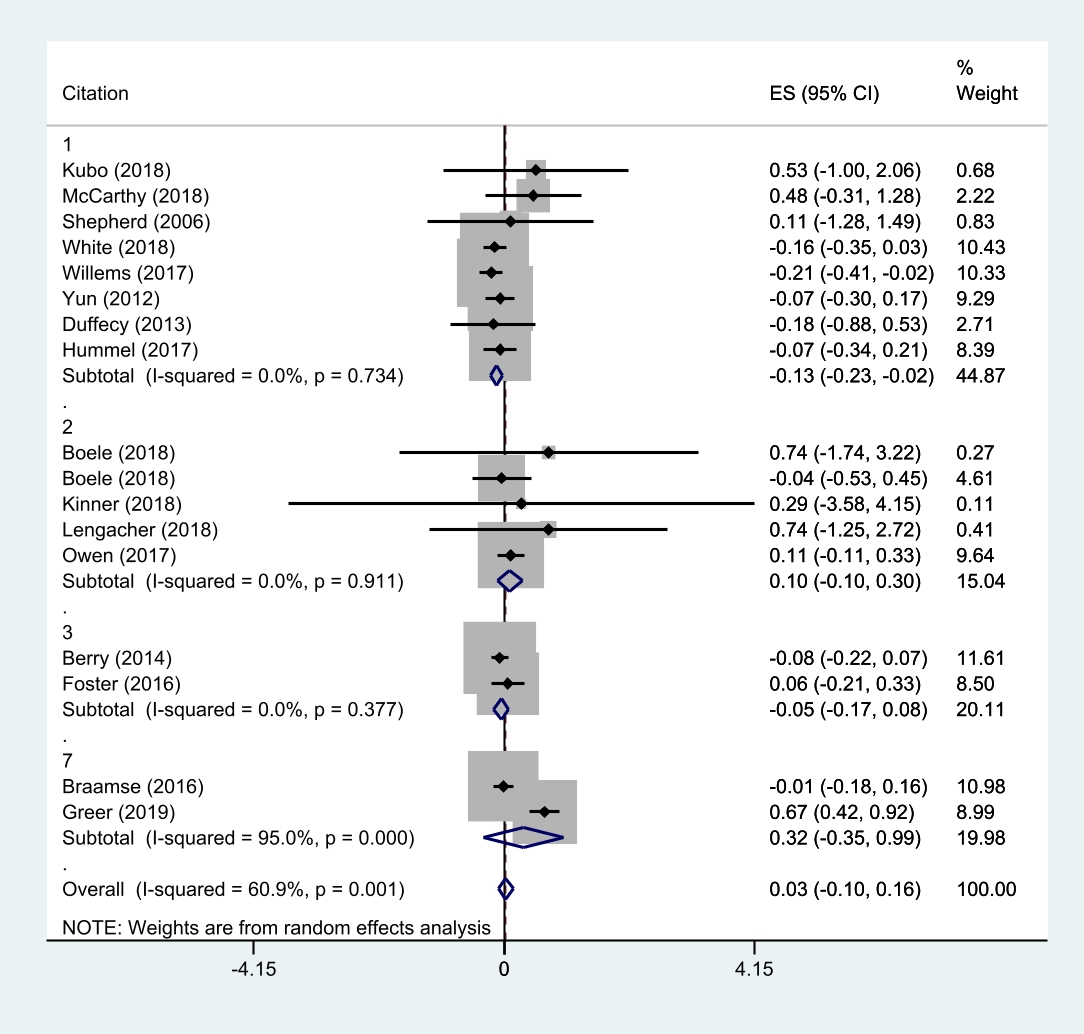


**Forest plot of studies reported on intervention effects on depression (time-varying effect; 1=Immediate, 2=interim, 3=short-term).**


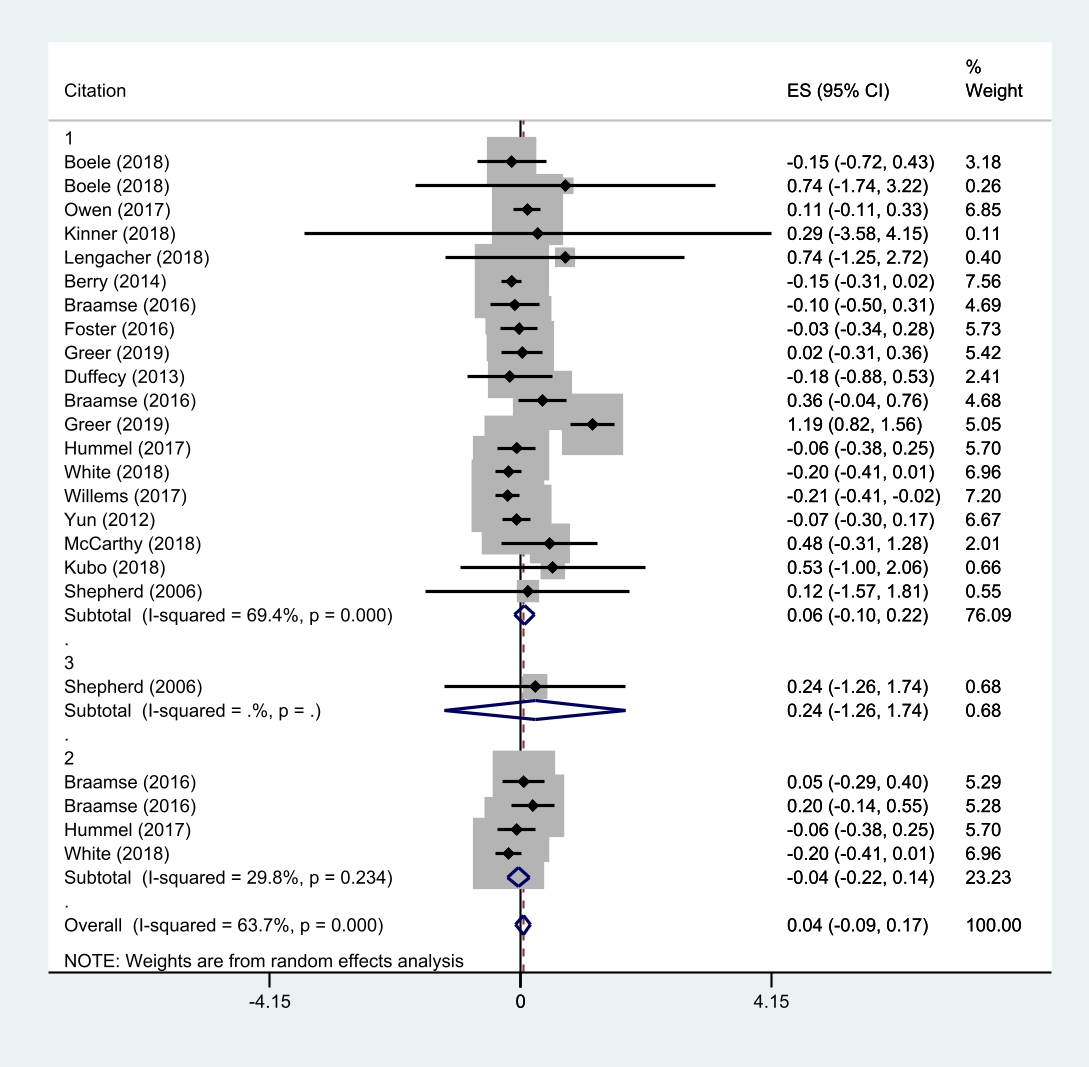


**Forest plot of studies reported on intervention effects on anxiety (overall effect, outcome measures: 1: HADS-anxiety subscale; 6: SSATI; 7: mixed).**


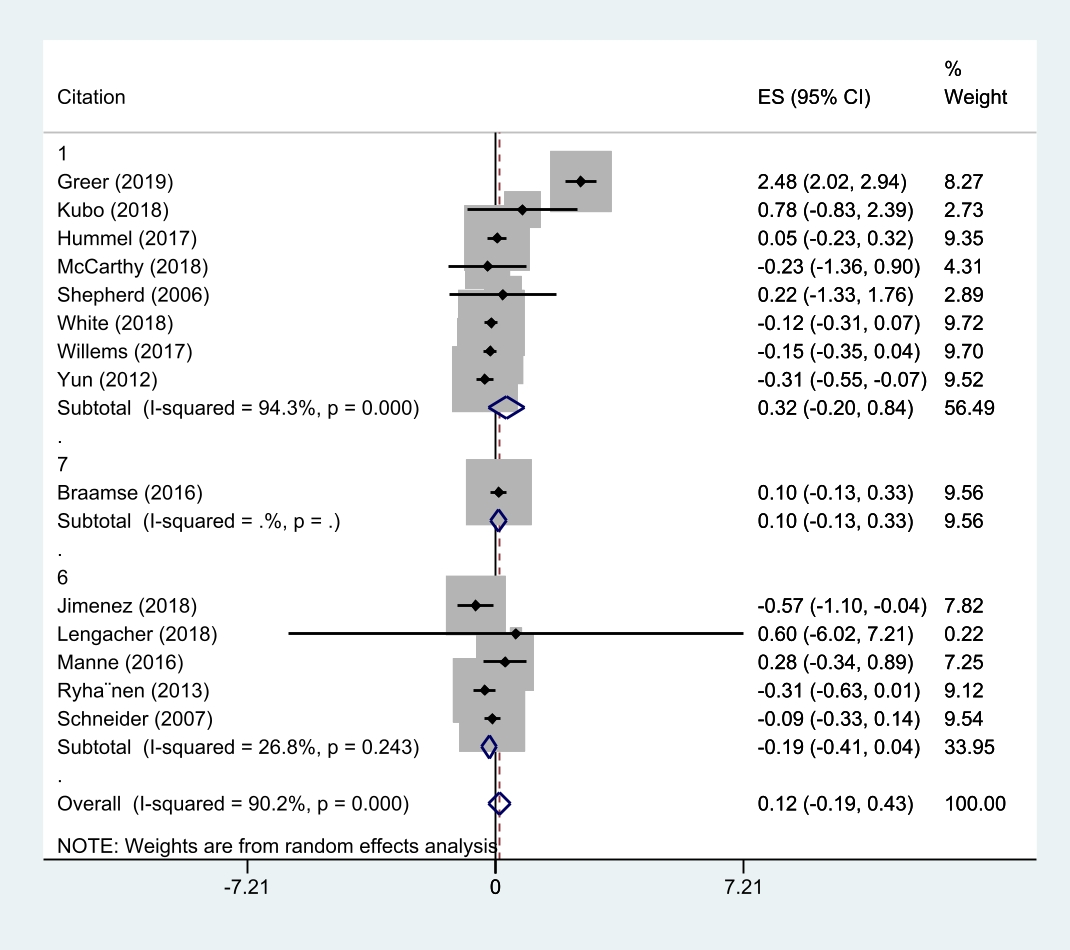


**Forest plot of studies reported on intervention effects on anxiety (time-varying effect, 1=Immediate, 2=interim, 3=short-term).**


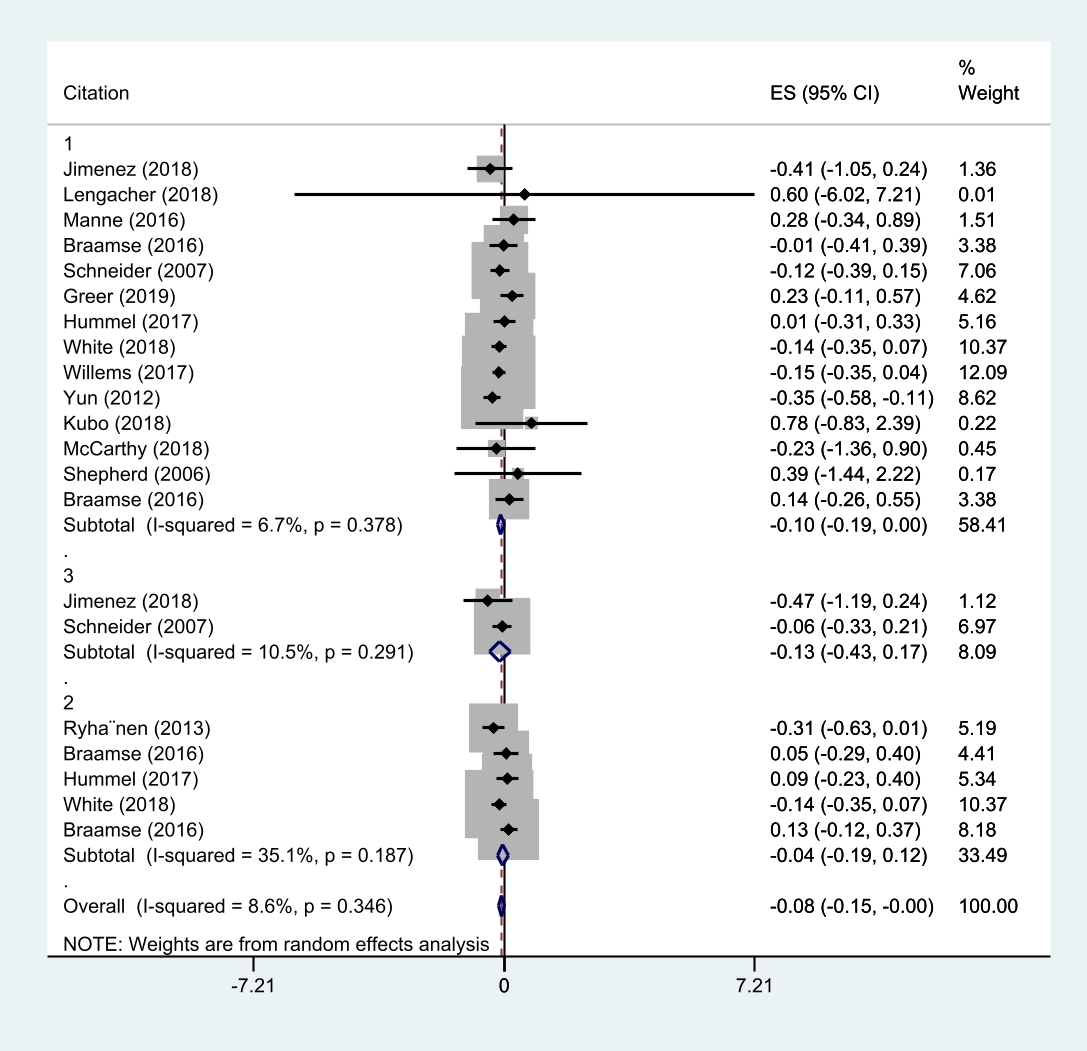


**Forest plot of studies reported on intervention effects on distress (overall effect).**


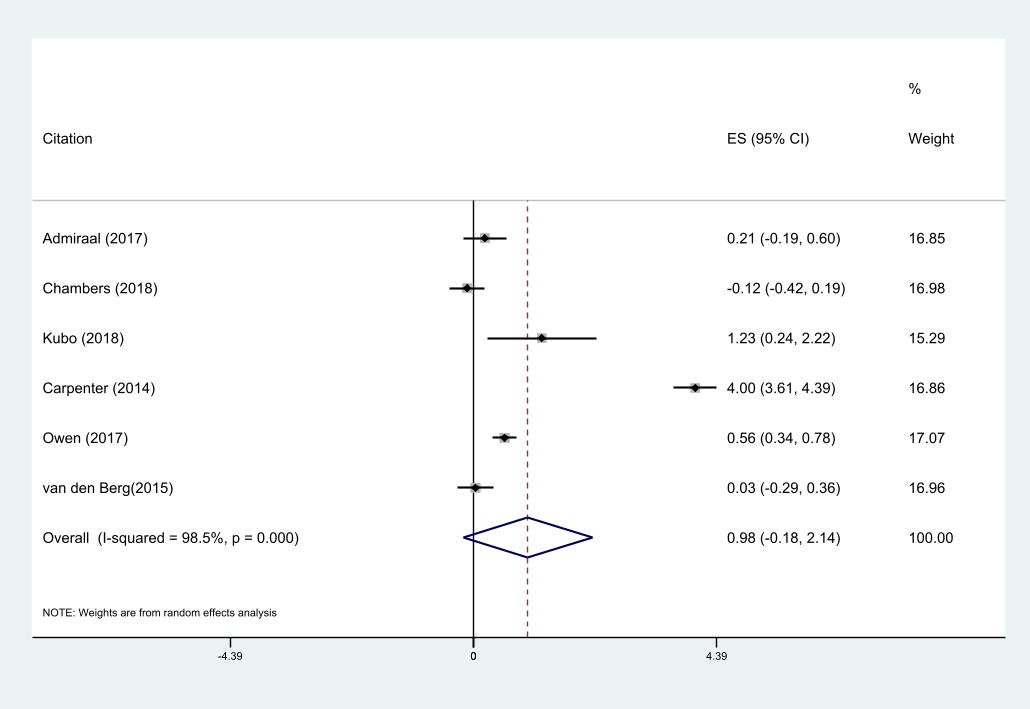


**Forest plot of studies reported on intervention effects on distress (time varying effect; 1=Immediate)**


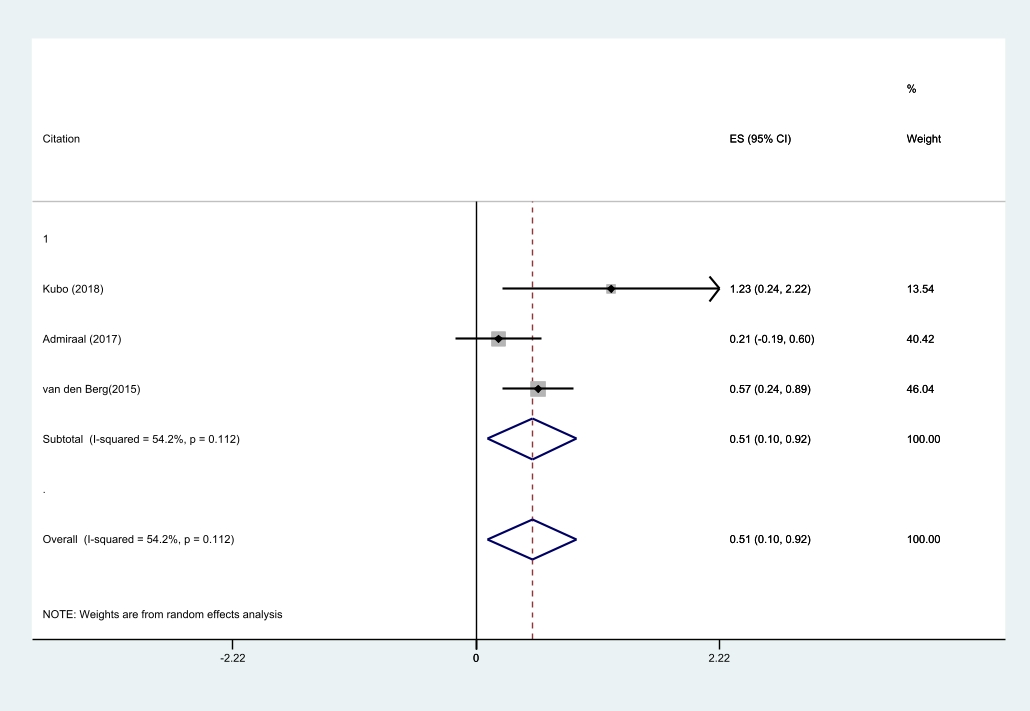


**Forest plot of studies reported on intervention effects on self-efficacy (overall effect).**


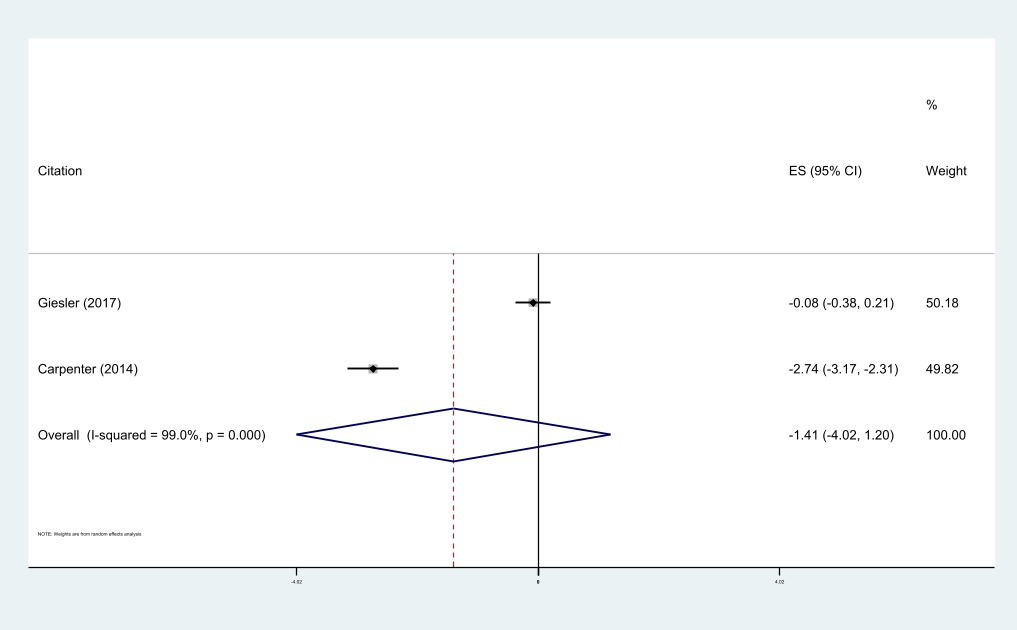


**Forest plot of studies reported on intervention effects on self-efficacy (time-varying effect, 1=Immediate).**


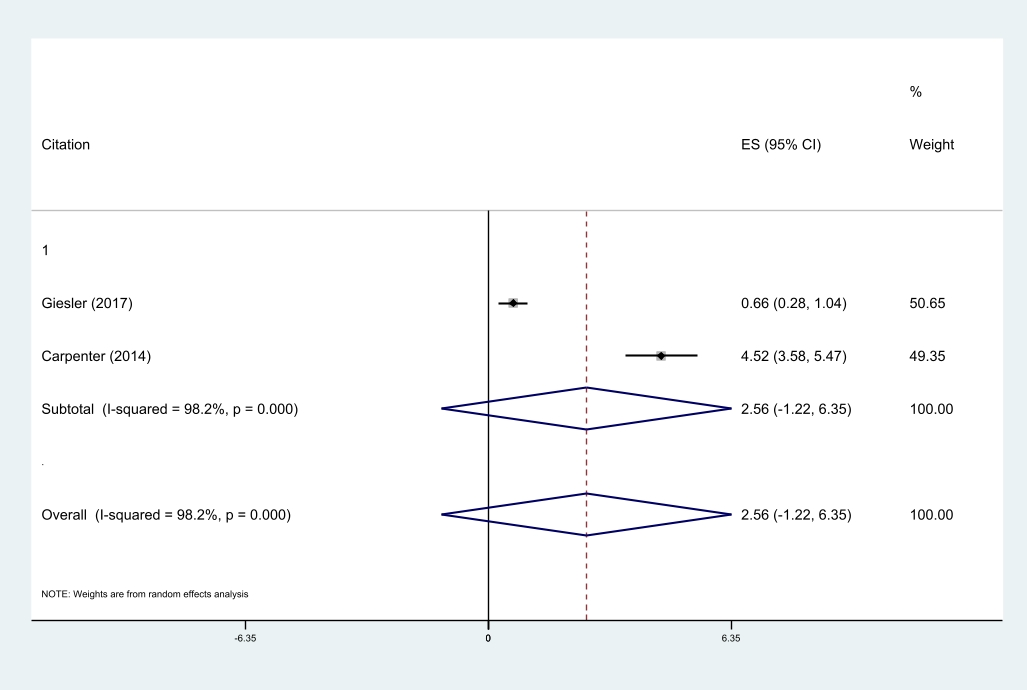

Supplement: Multimedia Appendix 3 [file cancer_v10i1e46116_app3.docx]
