# Supplementary material for: Digital Health Psychosocial Intervention in Adult Patients With Cancer and Their Families: Systematic Review and Meta-Analysis
Source: JMIR Cancer. 2024 Feb 5;10:e46116. doi: 10.2196/46116 (PMC10877499; doi:10.2196/46116)
Supplement: Multimedia Appendix 4 [file cancer_v10i1e46116_app4.docx]

Funnel plot of the individual study effect sizes plotted for quality of life in patient outcomes (overall effect)

Funnel plot of the individual study effect sizes plotted for quality of life in patient outcomes (time varying effect)

Funnel plot of the individual study effect sizes plotted for anxiety and depression measured by HADS total score in patient outcomes (overall effect)

Funnel plot of the individual study effect sizes plotted for Anxiety and depression measured by HADS total score in patient outcomes (time-varying effect)

Funnel plot of the individual study effect sizes plotted for depression in patient outcomes (overall effect)

Funnel plot of the individual study effect sizes plotted for depression in patient outcomes (time varying effect)

Funnel plot of the individual study effect sizes plotted for Anxiety in patient outcomes (overall effect)

Funnel plot of the individual study effect sizes plotted for anxiety in patient outcomes (time varying effect)

Funnel plot of the individual study effect sizes plotted for distress in patient outcomes (overall effect)

Funnel plot of the individual study effect sizes plotted for distress in patient outcomes (time varying effect)

Funnel plot of the individual study effect sizes plotted for self-efficacy in patient outcomes (overall effect)

Funnel plot of the individual study effect sizes plotted for self-efficacy in patient outcomes (time varying effect)
