# Supplementary material for: Digital Health Psychosocial Intervention in Adult Patients With Cancer and Their Families: Systematic Review and Meta-Analysis
Source: JMIR Cancer. 2024 Feb 5;10:e46116. doi: 10.2196/46116 (PMC10877499; doi:10.2196/46116)

Forest plot for the studies reported on intervention effects on anxiety in family members


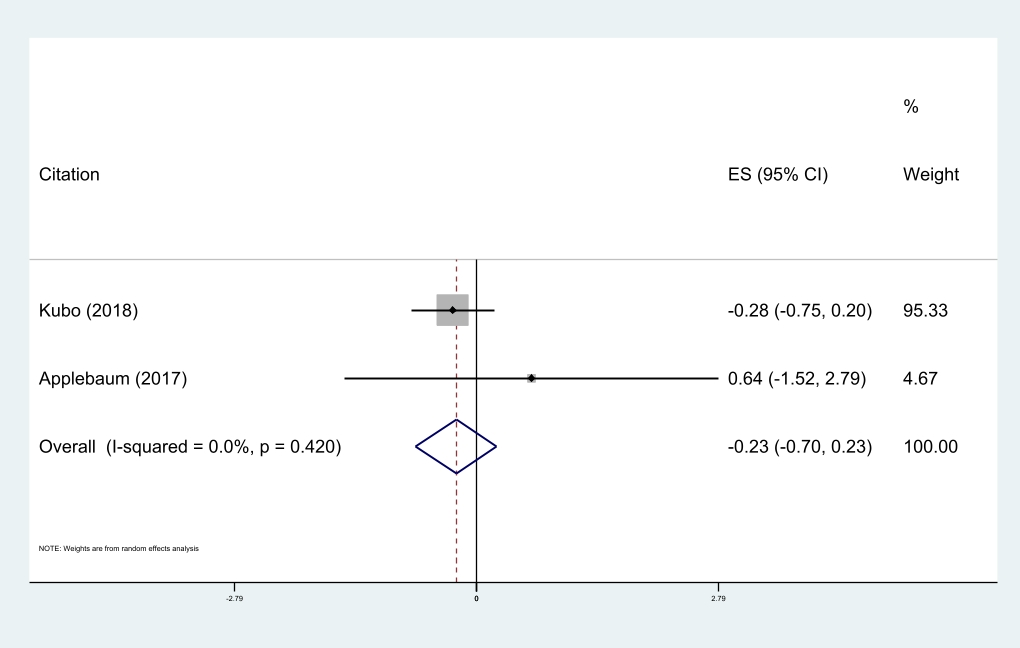


Forest plot for the studies reported on intervention effects on depression in family members


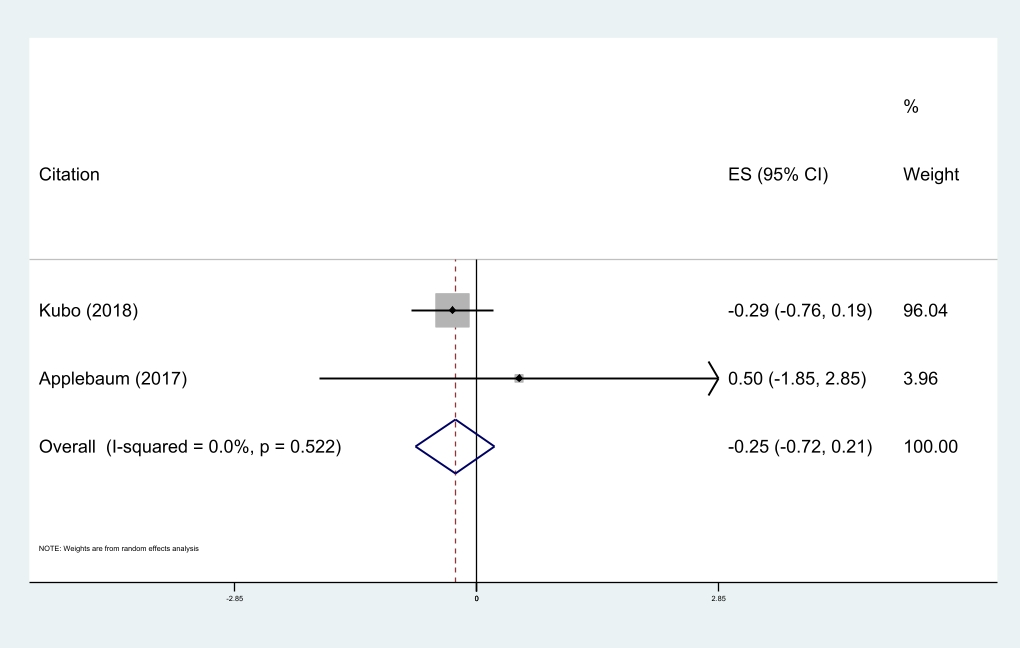

Supplement: Multimedia Appendix 5 [file cancer_v10i1e46116_app5.docx]
