# Supplementary material for: Digital Health Psychosocial Intervention in Adult Patients With Cancer and Their Families: Systematic Review and Meta-Analysis
Source: JMIR Cancer. 2024 Feb 5;10:e46116. doi: 10.2196/46116 (PMC10877499; doi:10.2196/46116)
Supplement: Multimedia Appendix 6 [file cancer_v10i1e46116_app6.docx]

Funnel plot of the individual study effect sizes plotted for depression in family member outcomes

Funnel plot of the individual study effect sizes plotted for anxiety in family member outcomes
